# Dissolved storage glycans shaped the community composition of abundant bacterioplankton clades during a North Sea spring phytoplankton bloom

**DOI:** 10.1186/s40168-023-01517-x

**Published:** 2023-04-17

**Authors:** Chandni Sidhu, Inga V. Kirstein, Cédric L. Meunier, Johannes Rick, Vera Fofonova, Karen H. Wiltshire, Nicola Steinke, Silvia Vidal-Melgosa, Jan-Hendrik Hehemann, Bruno Huettel, Thomas Schweder, Bernhard M. Fuchs, Rudolf I. Amann, Hanno Teeling

**Affiliations:** 1 Max Planck Institute for Marine Microbiology, Celsiusstraße 1, 28359 Bremen, Germany; 2 Alfred Wegener Institute for Polar and Marine Research, Biologische Anstalt Helgoland, P.O. Box 180, 27483 Helgoland, Germany; 3 Alfred Wegener Institute for Polar and Marine Research, Hafenstraße 43, 25992 List/Sylt, Germany; 4 Alfred Wegener Institute for Polar and Marine Research, Klußmannstraße 3, 27570 Bremerhaven, Germany; 5 Center for Marine Environmental Sciences, MARUM, University of Bremen, Leobener Straße 8, 28359 Bremen, Germany; 6 Max Planck Genome Centre Cologne, Carl Von Linné-Weg 10, 50829 Cologne, Germany; 7 Institute of Pharmacy, University of Greifswald, Felix-Hausdorff-Straße 3, 17489 Greifswald, Germany; 8 Institute of Marine Biotechnology, Walther-Rathenau-Straße 49a, 17489 Greifswald, Germany

**Keywords:** Algal bloom, Algal polysaccharide, Alpha-glucan, Bacterioplankton, Bacteroidota, Beta-glucan, Helgoland Roads LTER, Laminarin

## Abstract

**Background:**

Blooms of marine microalgae play a pivotal role in global carbon cycling. Such blooms entail successive blooms of specialized clades of planktonic bacteria that collectively remineralize gigatons of algal biomass on a global scale. This biomass is largely composed of distinct polysaccharides, and the microbial decomposition of these polysaccharides is therefore a process of prime importance.

**Results:**

In 2020, we sampled a complete biphasic spring bloom in the German Bight over a 90-day period. Bacterioplankton metagenomes from 30 time points allowed reconstruction of 251 metagenome-assembled genomes (MAGs). Corresponding metatranscriptomes highlighted 50 particularly active MAGs of the most abundant clades, including many polysaccharide degraders. Saccharide measurements together with bacterial polysaccharide utilization loci (PUL) expression data identified *β*-glucans (diatom laminarin) and *α*-glucans as the most prominent and actively metabolized dissolved polysaccharide substrates. Both substrates were consumed throughout the bloom, with *α*-glucan PUL expression peaking at the beginning of the second bloom phase shortly after a peak in flagellate and the nadir in bacterial total cell counts.

**Conclusions:**

We show that the amounts and composition of dissolved polysaccharides, in particular abundant storage polysaccharides, have a pronounced influence on the composition of abundant bacterioplankton members during phytoplankton blooms, some of which compete for similar polysaccharide niches. We hypothesize that besides the release of algal glycans, also recycling of bacterial glycans as a result of increased bacterial cell mortality can have a significant influence on bacterioplankton composition during phytoplankton blooms.

Video Abstract

**Supplementary Information:**

The online version contains supplementary material available at 10.1186/s40168-023-01517-x.

## Background

The carbon cycle is the largest biogeochemical cycle. Every year, more than 100 Gt of carbon are fixed by photosynthesis in about equal amounts by terrestrial plants and marine algae [23]. Marine planktonic microalgae (phytoplankton) fix about 45–50 Gt annually [21], of which diatoms alone might fix up to 20 Gt [53]. This carbon fixation climaxes during phytoplankton blooms, which can occur in the presence of sufficient solar irradiance and inorganic nutrients. Phytoplankton blooms can reach massive scales that are only visible in entirety by satellite, but are also usually rather short-lived. Bloom termination is often a result of compounding effects, such as self-shading, nutrient exhaustion, viral infections, parasitism by marine fungi, oomycetes and algicidal bacteria, and grazing by protists (e.g., flagellates, ciliates) and invertebrate metazoans (e.g., copepods) [13, 51, 80]. During blooms, copious amounts of organic material are released to the surrounding seawater, either via algal exudation or cell death. This fuels the pools of marine dissolved and particulate organic matter (DOM, POM), a substantial portion of which is rapidly remineralized by marine heterotrophic bacteria in surface waters.

Bloom-associated bacteria have co-evolved with photosynthetic microalgae since the latter emerged during the Precambrian Proterozoic period roughly two billion years ago [69], including the more recent diatoms that emerged during the Permian–Triassic extinction event about 250 million years ago [8]. These bacteria constitute tight-knit communities that collectively decompose algal biomass. Many partaking species are outright specialists that target only specific classes of algal biomass, a strategy which minimizes competition and concomitantly maximizes efficiency (resource partitioning) [22].

Polysaccharides (glycans) represent a major class of algal biomass, used for instance as intracellular energy storage or found in cell matrices and cell walls. Total contents depend on microalgal species and physiological state and can reach up to about 75% of the dry weight [59]. In addition, algae exudate glycans into the surrounding seawater, some of which assemble into transparent exopolymer particles (TEP). Many algal polysaccharides are anionic, often by sulfation, and have no counterparts in terrestrial plants. Simple algal polysaccharides consist of a sole monosaccharide and few linkage types, for example, laminarin, a helical polysaccharide composed of glucose monomers with a *β*-1,3-linked backbone and occasional *β*-1,6-linked branches. Laminarin serves as storage of photoassimilated glucose in diatoms and therefore is one of the most abundant polysaccharides on Earth [7]. Other algal polysaccharides are structurally more complex and involve numerous monosaccharides, linkage types, and secondary modifications. Knowledge on respective structures is sparse, in particular for planktonic microalgae (for diatoms, see [29]).

Polysaccharide decomposition requires various specifically adapted carbohydrate-active enzymes (CAZymes) belonging to different glycoside hydrolase (GH), polysaccharide lyase (PL), and carbohydrate esterase (CE) families [12]. In polysaccharide-degrading bacteria, genes for decomposition and uptake of dedicated polysaccharides are usually organized in operon-like polysaccharide utilization loci (PULs) that allow inferences about the chemical nature of the target polysaccharide substrate. PUL genes can account for a considerable proportion of genes in specialized bacterial clades (e.g., [44]). This constitutes a considerable genetic investment, which entails that resource partitioning is particularly pronounced among polysaccharide-degrading bacteria.

We have analyzed the response of planktonic bacteria (bacterioplankton) to spring phytoplankton blooms at Helgoland Roads in the German Bight of the North Sea since 2009 [25, 48, 71, 72]. Suitable conditions for these mostly diatom-dominated blooms usually occur from mid-March to the beginning of April. Initially, inorganic nutrients are usually aplenty and predator abundances are low, which allows algae and bacteria to grow with few restrictions. This largely bottom-up controlled phase is often characterized by swift successions of distinct algae and bacterioplankton taxa [72]. The latter usually represent about > 99% of the total bacteria in the water column [33]. As blooms progress, parasites and predators start to catch up. Top-down pressure from infections and grazing increases, and, together with nutrient depletion, ultimately, these blooms are terminated after a few weeks [36, 39, 64]﻿.

Phytoplankton blooms are highly dynamic events. Substantial changes in both algae and bacterioplankton community composition can happen within a day or two. Frequent sampling is thus required in order to disentangle what drives bacterioplankton dynamics. Highly resolved bacterioplankton community composition analysis is also required, since some clades comprise species with considerable functional diversity, whereby the genus *Polaribacter* is a prime example [6]. This diversity is usually not well addressed with partial 16S rRNA gene amplicon sequencing in combination with short-read-based metagenomics — methods that were common until recently. Finally, high time-resolution deep expression analysis is a precondition to capture key metabolic processes of distinct clades over a bloom’s progression.

Here, we present a study of the 2020 spring phytoplankton bloom at Helgoland Roads. We sampled the complete spring bloom period over 90 days and sequenced bacterioplankton metagenomes and transcriptomes at 30 dates. These were complemented by algae and bacteria diversity and abundance data, copepod and flagellate abundance data, physicochemical data and measurements of mono- and polysaccharide concentrations. These data allowed us to investigate (i) which dissolved polysaccharides are available to and are preferentially consumed by abundant, active bacterioplankton clades, (ii) how the availability and consumption of polysaccharides changed over time, and (iii) whether these processes might be influenced by changes in algae composition and algae and flagellate abundances.

## Results

### The 2020 Helgoland spring phytoplankton bloom was biphasic

**Fig. 1:**
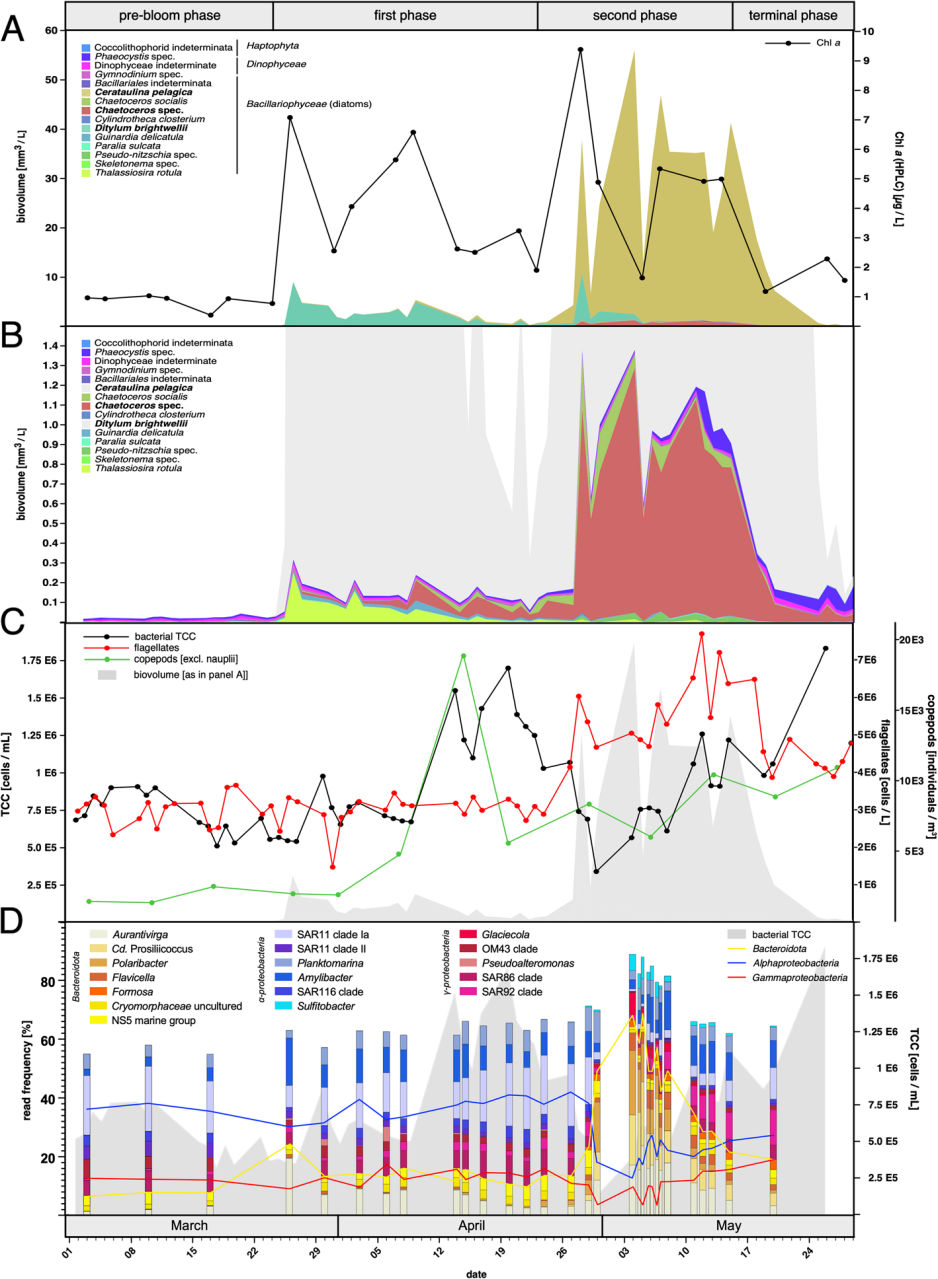
Major phases of the spring phytoplankton bloom at Helgoland Roads during March to May 2020. **A** Biovolumes of abundant phytoplankton taxa and chlorophyll *a* concentrations as measured by HPLC (black line). **B** Biovolumes of less abundant algal taxa. **C** DAPI-based bacterial total cell counts (TCC; black line), flagellate counts (red line), and counts of copepods (excluding *Copepoda nauplii*; green line). Estimated phytoplankton biovolumes are plotted as a gray area in the background. **D** Abundances of the 18 most prominent bacterial clades as assessed by metagenomic 16S rRNA gene frequencies. The sum of bacteroidetal, alpha-, and gammaproteobacterial clades among these top 18 clades is depicted by lines. Bacterial total cell counts are represented as gray area in the background

The 2020 spring phytoplankton bloom at Helgoland Roads started around March 24 and lasted until the end of May. It consisted of two distinct phases with an inflection point around April 23 (Fig. 1). The first bloom phase started with a sevenfold increase in chlorophyll *a* from ~ 1 to ~ 7 µg per L within just 2 days and went through a total of three maxima. The combined algal biovolume during this phase was dominated by the large centric diatom species *Ditylum brightwellii* (Fig. 1A), with minor contributions from *Phaeocystis* sp. (*Haptophyta*, class *Prymnesiophyceae*), unspecified *Dinophyceae*, and *Cerataulina pelagica*, *Chaetoceros* sp., *Guinardia delicatula*, and *Thalassiosira rotula* centric diatoms (Fig. 1B). *D. brightwellii* numbers declined towards the end of the first phase with chlorophyll *a* declining to ~ 2 µg per L, passing into the second phase. This phase was characterized by rapid proliferation of *Chaetoceros* sp. and in particular large *C. pelagica* centric diatoms, which resulted in an initial chlorophyll *a* increase from below 2 to above 9 µg per L within 5 days. During the second phase, also *Phaeocystis* sp. and *Dinophyceae* numbers started to increase, but did not reach notable proportions of the total algal biovolume (Additional file 2). At the same time, the inorganic nutrients silicate, phosphate, ammonium, and nitrate became scarce (Fig. S1 C, D in Additional file 1; Additional file 2), and the bloom went into decline from about mid-May on. During this terminal phase, *Dinophyceae* numbers decreased less rapidly than diatom numbers, probably because the former do not depend on the silicate which the latter need for frustule formation. At the end of May, the bloom was over, and algal cell numbers were almost down to pre-bloom levels.

During the first bloom phase, flagellate numbers remained low at an almost constant 2 to 3 × 10^6^ per L, indicating substantial grazing pressure from higher trophic levels (Fig. 1C). Microscopic observations confirmed a pronounced copepod bloom during the second half of this phase that was dominated by *Paracalanus* species (Additional file 3). The copepod bloom started around April 1, and the highest numbers were counted on April 15 using an about weekly counting interval (Fig. 1C). The increase in copepod numbers was accompanied by a pronounced increase in bacterioplankton with total cell counts (TCC) from around 0.5 to 1.7 × 10^6^ per mL (Fig. 1C). Afterwards, both algal biomass and copepod numbers subsided, while flagellate numbers started to increase, and bacterial TCC to drop to about 10^6^ per mL at the bloom’s inflection point (Fig. 1C). The second bloom phase was characterized by much higher overall algae numbers. At first, bacterioplankton continued to decrease down to about 0.3 × 10^6^ per mL and then increased again to finally reach about 1.8 × 10^6^ per mL on May 26. Likewise, flagellate abundances increased throughout most of the second bloom phase until a decrease towards the bloom’s end, while copepod numbers increased only little throughout the second bloom phase (Fig. 1C).

### The second bloom phase was triggered by an influx of nutrient-rich coastal water

Helgoland is located on the continental shelf with water depths up to ~ 56 m. The water body at the shallower (6 to 10 m depth) long-term ecological research (LTER) site ‘Kabeltonne’ at Helgoland Roads (54° 11.3′ N, 7° 54.0′ E) is subject to tidal range and usually well-mixed [78]. There are competing influences from oceanic and coastal water masses that can disturb the system to varying extents. Still, the water regime can be relatively stable for longer periods of time.

The dominating wind directions in the German Bight are northwest and southwest. Due to the Coriolis pseudo force, these winds suppress spreading of coastal waters from the south offshore. However, when northeasterly to easterly winds prevail for some days, coastal water masses and plumes from the Elbe and Weser river estuaries (~ 50 km distance) can reach Helgoland. These waters are usually characterized by lower salinities and higher concentrations of inorganic nutrients, resulting in a drop of salinity and in an increase of nutrients (e.g., [15, 67]). In particular, nitrate is a suitable proxy for riverine inputs in the southern North Sea due to extensive agriculture in the North German Plain.

On April 1/2 and at the onset of the second phase on April 22/23, inorganic ammonium and phosphate concentrations spiked almost threefold to about 5.1–6.8 µM and 1.6–1.8 µM, respectively. Both spikes were accompanied by increases in silicate concentrations, and drops in salinities from about 33.8 to 31.6 and 32.1 to 30.4, respectively (Fig. S1 A, C in Additional file 1), indicating incursions of nutrient-rich coastal waters. The second influx event was accompanied by a spike in nitrate concentration of 33.8 µM at April 21, representing the highest nitrate concentration measured during the sampling campaign (Fig. S1C in Additional file 1). Wind directional data supported an influx of coastal waters, since moderate northeasterly to easterly winds dominated from April 19 to 24 (Fig. S2 in Additional file 1, Additional file 4). The influx of fresh nutrients at the bloom’s inflection point together with continuously increasing water temperatures likely set the stage for the second phase of the bloom to take place. At the same time, species abundances around the bloom's inflection point were likely influenced by coastal water intrusions.

### Metagenome 16S rRNA gene frequencies revealed that few flavobacterial clades dominated the second bloom phase

Unassembled metagenome reads contained 325,737 near full-length 16S rRNA gene sequences (average: 1269 bp) that were used for diversity analyses (Fig. 1D, Additional files 5, 6). Among *Alphaproteobacteria*, SAR11 Ia clade sequences dominated during the pre- and first bloom phases, reaching up to 22.1% on March 10 and 21.4% on April 20. Likewise, SAR11 clade II sequences were confined to the pre- and first bloom phases and peaked with 5.1% also on March 10. In contrast, *Amylibacter* sequences were detected during all bloom phases reaching up to 16.2% on March 26 and 13.3% on May 7 (9 PM sample). A similar pattern was found for *Planktomarina* with a maximum of ~ 11% on April 30. *Sulfitobacter* sequences were associated with the second and terminal bloom phases and reached up to ~ 8%, while SAR116 sequences never exceeded ~ 5% at any time point.

Sequences of abundant *Bacteroidota* affiliated with well-known bloom-associated *Flavobacteriia* clades at Helgoland, such as *Aurantivirga* [48], *Cd. * Prosiliicoccus [25], and *Polaribacter* [6]*.* Flavobacterial clades ramped up at the bloom’s inflection point and dominated in the second bloom phase, topping out at 56% on May 4. *Flavobacteriia* relative numbers declined after May 8 during the bloom’s terminal phase. The most abundant flavobacterial clade, *Aurantivirga,* peaked with 19% of the bacterial relative abundance on March 26 during the first phase and with ~ 23% on May 5 during the second phase. In contrast, *Polaribacter* were only detected in the second bloom phase, peaking with 21.7% relative abundances on May 5. *Flavicella* members were present during the second and terminal bloom phases and reached up to 6.5% on May 6. The NS4, NS5, NS2b, NS3a, and NS9 clades [2] occurred during the pre- and first bloom phases. Sequences of other *Bacteroidota* clades such as *Formosa*, *Fluviicola*, *Marinoscillum*, *Aquibacter*, and uncultured *Cryomorphaceae* were found during all bloom phases with proportions below 5%.

The most prominent *Gammaproteobacteria* were members of the SAR92 clade, in particular during the terminal bloom phase with up to ~ 12% relative abundance on May 20. In contrast, SAR86 sequences were present mostly during the pre- and second bloom phases and during the terminal phase after May 8. Members of the OM182 clade followed a similar pattern, but relative abundances never exceeded ~ 3%. *Methylophilaceae* (OM43 clade, reclassified from *Beta-* to *Gammaproteobacteria* in GTDB [58]) were also restricted to the pre- and first bloom phases. *Glaciecola* sequences were constricted to the second bloom phase with a maximum of ~ 8% on May 4. *Pseudoalteromonas* 16S rRNA gene sequences were found in the first bloom phase with a maximum of ~ 5% on April 6. Sequences of *Luminiphilus* OM60 (NOR5 clade) were found in all bloom phases but with low frequencies. In contrast, *Cd.* Thioglobus (SUP05 clade) sequences were confined to the pre-bloom phase before March 26.

Sequences of actinobacterial *Cd.* Actinomarina were restricted to the pre- and first bloom phases with a maximum of ~ 5% relative abundance on March 10. Marine group II *Archaea* sequences were low in abundance throughout the bloom, with highest relative abundances of ~ 11% during the pre-bloom phase on March 26.

### Abundant clades were confirmed by CARD-FISH analyses

**Fig. 2:**
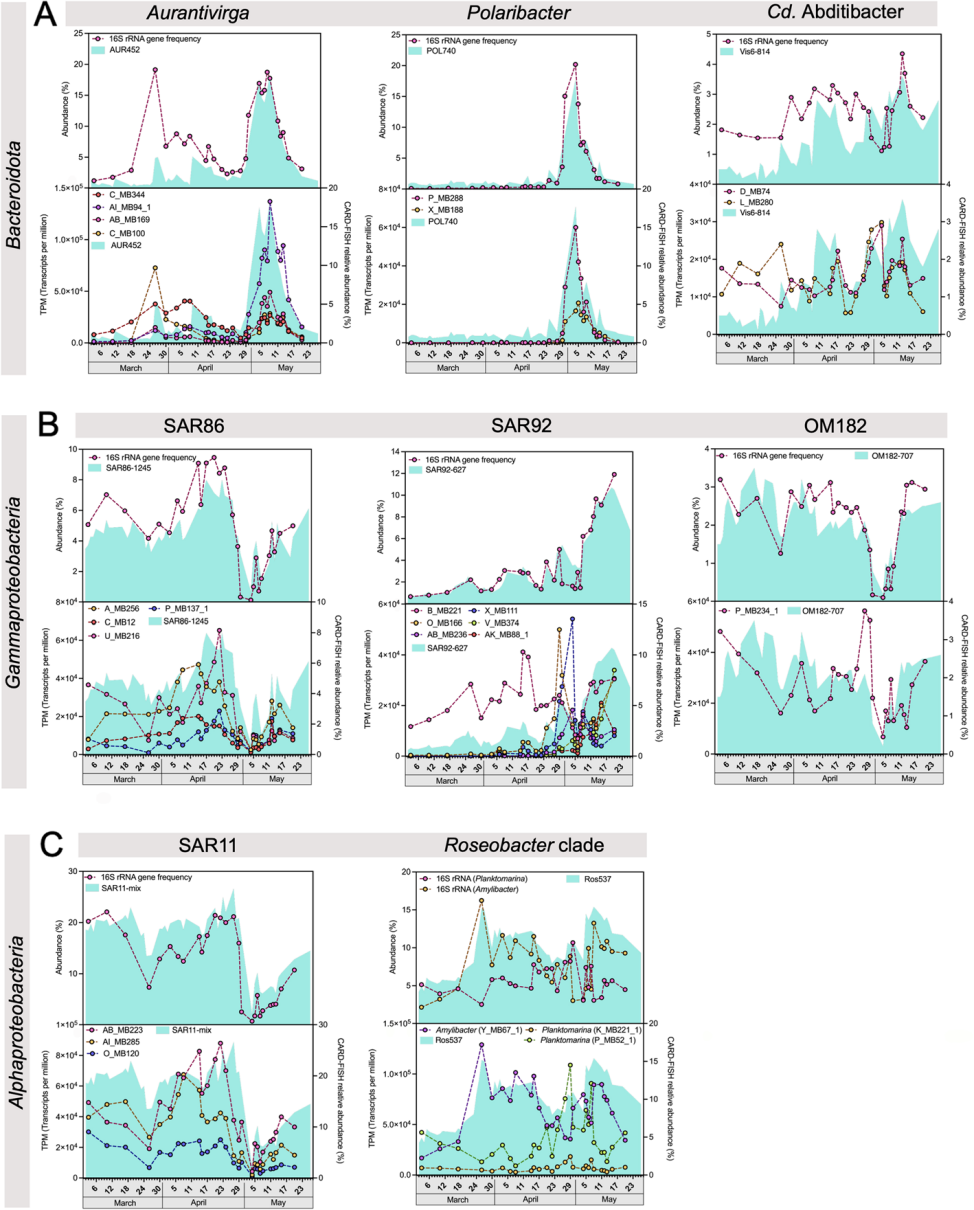
Upper graphs: Abundance of various prominent clades as assessed by CARD-FISH (turquoise areas) and by metagenomic 16S rRNA gene frequencies (lines). Lower graphs: Abundances as assessed by CARD-FISH (turquoise areas) and expression patterns of corresponding metagenome-assembled genomes as assessed by metatranscriptomics (lines) expressed as transcripts per million (TPM). **A** *Bacteroidota*: *Aurantivirga* (probe AUR452), *Polaribacter* (probe POL740) and *Cd*. Abditibacter (probe Vis6-814). **B** *Gammaproteobacteria*: SAR86 clade (probe SAR86-1245), SAR92 clade (probe SAR92-627), and OM182 clade (probe OM182-707). **C** *Alphaproteobacteria*: SAR11 clade (probe SAR11-mix) and RCA clade *Planktomarina* and *Amylibacter* (probe Ros537)

Microscopic cell counting with fluorescently labeled CARD-FISH (catalyzed reporter deposition-fluorescence in situ hybridization) probes (Additional file 7) confirmed *Aurantivirga* (probe AUR452) and *Polaribacter* (probe POL740) as the dominant clades within the *Bacteroidota,* with maximum relative abundances of 18.3% (112,000 cells/mL) and 17.7% (100,000 cells/mL) on May 8 and 4, respectively (Fig. 2A). Overall abundances of both clades were highest during the second bloom phase, but seldom exceeded 5% during other phases. This was the case when *Aurantivirga* peaked to slightly above 5% relative abundance on March 26/27 (~ 27,000 cells/mL). This peak coincided with the initial *D. brightwellii* peak, suggesting a rapid response of *Aurantivirga* to the proliferation of this diatom*. Cd. * Abditibacter ([28], probe Vis6-814), on the other hand, had low overall abundances, ranging from 0.1 to 3.4% throughout the bloom. Towards the end of the second phase into the bloom’s terminal decline, relative abundances of dominating *Bacteroidota* declined, whereas those of the gammaproteobacterial SAR92 clade (probe SAR92-627) gradually increased to a maximum of 10.7% (105,000 cells/mL) on May 19 (Fig. 2B). Members of the gammaproteobacterial SAR86 clade (probe SAR86-1245) exhibited higher relative abundances during the first than the second bloom phase, but values never exceeded 8%. The gammaproteobacterial OM182 clade (probe OM182-707) showed an almost uniform distribution throughout the bloom, with values mostly ranging below 4.5%. Both SAR86 and OM182 relative abundances tanked below 1% on May 4 and recovered afterwards. Such a minimum was not observed for the SAR92.

Corroborating metagenome 16S rRNA gene frequencies, SAR11 (probe SAR11-mix) dominated during the pre-bloom and both major bloom phases with relative abundances ranging from 18.6% (127,000 cells/mL) on March 2 to 26.6% (286,000 cells/mL) on April 27 (Fig. 2C). Like the gammaproteobacterial SAR86 and OM182 clades, SAR11 also suddenly dropped to 2.1% relative abundance on May 4 with a slow recovery afterwards that lasted throughout the bloom’s terminal phase. Members of the abundant *Roseobacter* clade (probe Ros537) were more uniformly distributed, with peaks in relative abundance of around ~ 15% (~ 83,000 cells/mL) on March 26/27 (initial *D. brightwellii* peak) during the first bloom phase, and on May 8 during the second bloom phase. Abundant members comprised *Amylibacter* and *Planktomarina*, with *Amylibacter* being abundant throughout the bloom, and *Planktomarina* thriving during the second bloom phase (Fig. 2C).

### High-quality representative MAGs could be obtained for most abundant clades

Automatic binning of all 30 individual metagenomes yielded 10,950 initial bins and 11,071 bins after manual refinement (Additional file 8, Fig. S3A in Additional file 1). Of these, 1648 had > 70% completeness and < 5% contamination estimates (Fig. S3B in Additional file 1). Dereplication at 0.95 ANI reduced this set to 251 representative, non-redundant MAGs, 165 of which had > 90% completeness, and < 5% contamination estimates. According to MIMAG standards [10], 158 MAGs were classified as high quality. A total of 140 MAGs consisted of ≤ 10 contigs, and 76 even had a no predicted contamination, substantiating the dataset’s high quality (Fig. S3C in Additional file 1).

GTDB taxonomic affiliation revealed that 97.2% of the MAGs belonged to *Bacteria* and 2.8% to *Archaea*. On phylum level, *Proteobacteria* (48.6%) dominated, followed by *Bacteroidota* (36.3%). The remaining bacterial MAGs belonged to *Actinobacteriota*, *Verrucomicrobiota*, *Planctomycetota*, *Marinisomatota*, *Myxococcota*, and *Campylobacterota* (Additional file 9). These representative MAGs correlated well with the overall abundant clades detected by CARD-FISH as well as 16S rRNA gene frequencies.

### Few particularly active MAGs dominated overall expression

**Fig. 3:**
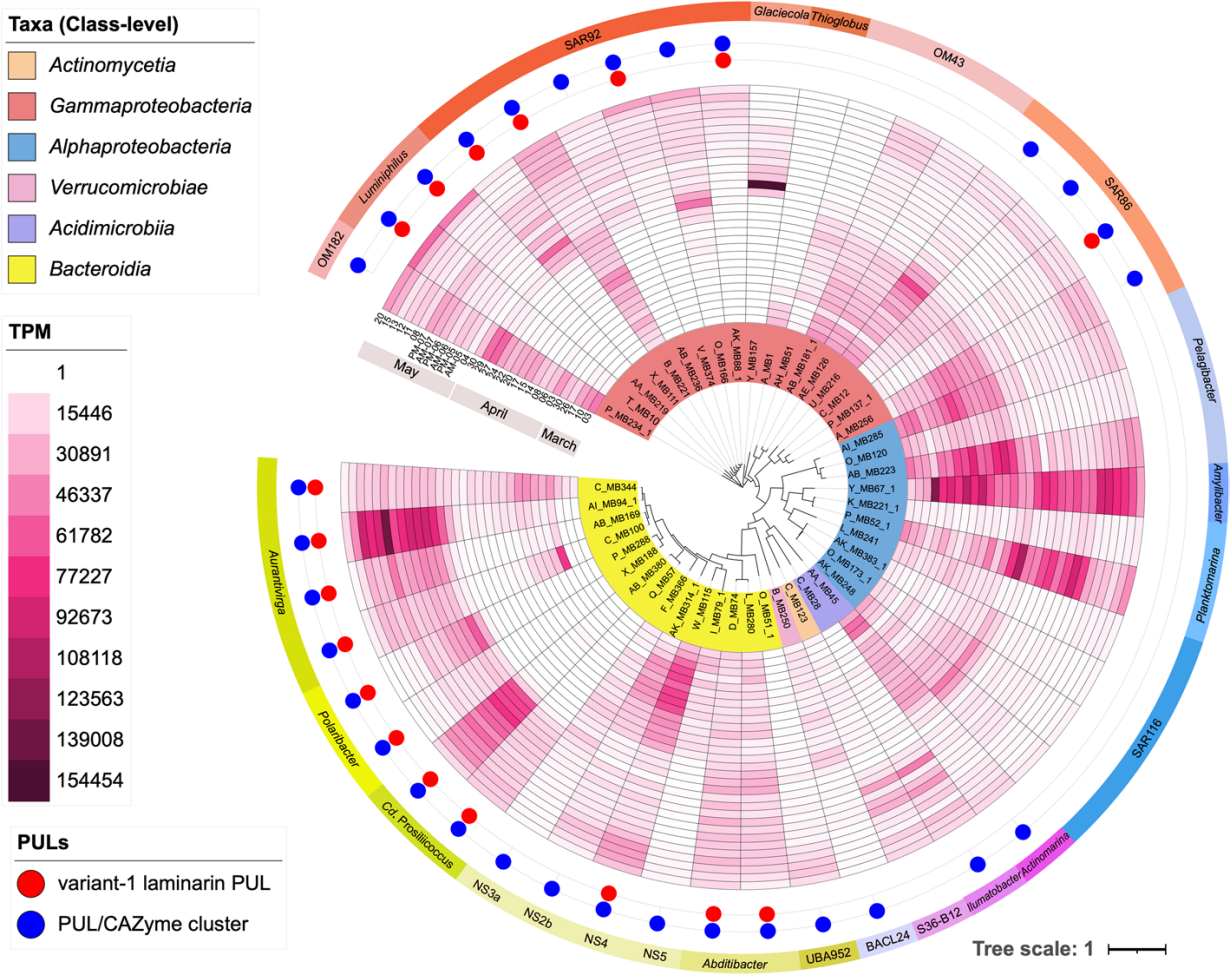
Phylogenomic tree of the top 47 expressed bacterial MAGs (created with *anvi-gen-phylogenomic-tree* of anvi’o v7) and their denominations along with their expression profiles. The expression is represented as heatmap of transcripts per million (TPM) along all sampling points from March to May 2020 (inside to outside). Genus-level clade affiliations are plotted at the outside. The outermost dots indicate the presence of predicted PULs (blue) and variant 1 laminarin PULs (red), respectively. Tree annotations were done with iTOL v6. Corresponding archaeal MAGs are depicted in Fig. S5 (Additional file 1)

Mapping of reads from all 30 transcriptomes onto the complete metagenome dataset recruited 71.5% of the transcripts, and onto the 251 representative MAGs 41.4% of the transcripts. During the pre-bloom, gammaproteobacterial and alphaproteobacterial MAGs dominated with 34.5% (349,824) and 32.1% (320,829) of the total transcripts per million (TTPM), respectively (Fig. S4 in Additional file 1). At the onset of the first bloom phase, relative activities of *Bacteroidia* and *Poseidonia* A (*Thermoplasmatota*) MAGs increased notably and peaked on March 26 during the first *D. brightwellii* peak with 36.3% (363,562) and 20.4% (203,861) of the TTPM, respectively. During the second bloom phase after April 27, relative activities of alphaproteobacterial MAGs decreased and reached a minimum of 18.0% (179,681) of the TTPM on May 13. Simultaneously, relative activities of *Bacteroidia* MAGs increased during the second bloom phase and even dominated a larger part of this phase with a maximum of 53.2% (532,164) of the TTPM on May 8 (Fig. S4 in Additional file 1; Additional file 10). The terminal phase lastly was characterized by a decrease in *Bacteroidota* and an increase in *Gammaproteobacteria* transcripts. As the bulk of metatranscriptome reads mapped to a small number of particularly active MAGs, we confined our analysis to the 50 topmost expressed MAGs representing 30.3% of all transcripts (Fig. 3; Fig. S5 in Additional file 1).

#### Bacteroidota

Ninety-one (36%) of the 251 representative MAGs affiliated with *Bacteroidota*, with 15 ranging among the 50 topmost expressed MAGs. *Aurantivirga* was the most active clade, with four MAGs accounting for up to 24.1% (241,055) of the TTPM. The most active of these MAGs (AI_MB94_1) exhibited highest expression during the second bloom phase after a steep rise from 1.0% (9958) of the TTPM on April 29 to 13.7% (136,921) of the TTPM on May 8. The second-most active *Aurantivirga* MAG (C_MB100) exhibited maximum expression of 7.3% (72,698) of the TTPM on March 26 during the *D. brightwellii* peak, indicating rapid response to proliferation of this diatom. Correlation with CARD-FISH data was high (*R* = 0.83; *p* = 1.03 E-7), confirming *Aurantivirga* as prominent bloom-associated clade (Fig. 2A).

The second-most expressed *Bacteroidota* clade was represented by two *Cd.* Prosiliicoccus MAGs. Their combined maximum expression accounted for up to 9.5% (95,261) of the TTPM, with one MAG (AB_MB380) alone accounting for 7.5% (74,669). Expression of both MAGs was negligible during the pre- and first bloom phases, but started to increase after April 29 and for AB_MB380 peaked on May 6 during the second bloom phase (Fig. 3). A similar pattern was observed for one of two notably expressed *Polaribacter* MAGs (P_MB288), with a peak on May 4 with 6.0% (60,030) of the TTPM. Information on other lower expressed *Bacteroidota* MAGs is included in Additional file 1.

Further lower expressed *Bacteroidota* MAGs included two *Cd.* Abditibacter MAGs and four MAGs from different NS clades. The *Cd.* Abditibacter MAGs exhibited almost uniform low expression throughout March to May. In contrast, MAGs affiliating with NS5 and NS3a marine groups exhibited higher expression during the first bloom phase, while MAGs affiliating with the NS4 and NS2b marine groups had higher expression before April 27 (first phase) and after May 12 (terminal phase) (Fig. 3).

#### Gammaproteobacteria

Eighteen out of 78 representative gammaproteobacterial MAGs were part of the 50 topmost expressed MAGs. A single *Glaciecola* MAG (Y_MB157) exhibited a pronounced peak in abundance and expression on May 4, making it the topmost expressed gammaproteobacterial clade with 15.4% (154,454) of the TTPM and 7.6% of relative 16S rRNA gene abundance. However, its peak expression lasted for only about a day, dropped to 2.2% afterwards, and subsequently, activity of *Glaciecola* Y_MB157 almost vanished with a mere 30 TPM left on May 20.

Six MAGs affiliating with SAR92 exhibited high expression in the second bloom phase except MAG B_MB221, which was notably expressed throughout the entire bloom. The five other SAR92 MAGs combined contributed up to 9.3% (93,093) of the TTPM, suggesting a relevance akin to that of abundant *Bacteroidota* clades. Two *Luminiphilus* (OM60/NOR5 clade) MAGs showed only little expression during the second bloom phase, with a notable increase on the last sampling date of the terminal phase (Fig. 3).

In contrast, SAR86 clade MAGs showed high expression mostly during the first bloom phase, during which all four respective MAGs combined accounted for 4.0% (39,694) to 11.5% (115,285) of the TTPM. Expression decreased considerably after April 29, reaching a minimum of only 0.8% (7,622) of the TTPM on May 4. Similar to SAR92, expression of SAR86 increased again after May 8. Also, MAGs affiliating with the OM43 clade exhibited a similar overall activity pattern.

#### ***Alphaproteobacteria﻿***

The 50 topmost expressed MAGs included ten alphaproteobacterial MAGs, with highest expression of three *Pelagibacter* (SAR11 clade) MAGs (AI_MB285, O_MB120, AB_MB223). Their collective expression ranged from 4.2% (42,104) to 16.4% (164,238) of the TTPM during the pre- and first bloom phases. Lower expression was observed for the related SAR116 clade, whose expression peaked during the first bloom phase (Fig. 3). An *Amylibacter* MAG (Y_MB67_1) showed high expression throughout the bloom with expression ranging from 1.7% (16,832) to 12.9% (128,976) of the TTPM. In contrast, the more active of two *Planktomarina* MAGs (P_MB52_1) was particularly active during the second bloom phase, with maximum expression of 10.9% (108,806) of the TTMP on April 29.

#### ﻿Other clades

Further highly expressed clades affiliated with *Actinobacteriota* (genera: *Actinomarina*, *Ilumatobacter*, *Cd*. Nanopelagicales) and *Verrucomicrobiota* (BACL24 clade). The top 50 expressed MAGs also contained three archaeal MAGs that all belonged to the *Poseidoniaceae* family and some further *Gammaproteobacteria* MAGs belonging to *Thioglobus* A (SUP05 clade) and the OM182 clade (Additional file 1).

### Dissolved polysaccharides were prime targets of abundant *Bacteroidota* and *Gammaproteobacteria*

**Fig. 4:**
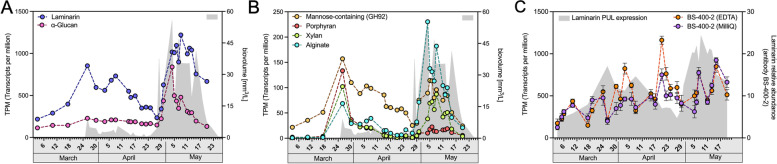
**A** Combined expression of PULs predicted to target the two most abundant polysaccharide substrates laminarin (GH149, GH17, GH16, GH30, GH158) and *α*-glucans (GH13, GH65, GH31). **B** Combined expression of PULs predicted to target less abundant polysaccharide substrates, such as mannose-rich polysaccharides (GH92), porphyran (GH29, GH86), xylan (GH43, GH10), and alginate (PL6, PL7, PL8, PL17). **C** Antibody-based (BS-400–2, specific for *β*-1,3-glucan) measurements of dissolved laminarin extracted from high-molecular-weight dissolved organic matter (HMWDOM) either with Milli-Q or EDTA (lines) as compared to the sum of expressed laminarin PULs in transcripts per million (TPM; gray area)

Thirty-two of the topmost 50 expressed MAGs featured expressed PULs/CAZyme clusters (Fig. 3). Summated expressions for each predicted substrate showed highest peak expression for PULs targeting laminarin, followed by *α*-glucans, alginate, *α*-mannose-, and xylan/xylose-containing polysaccharides as well as putative porphyran. The combined expression correlated well with algal biovolume estimates (*R*: 0.79; *p*: 1.13 E-6) (Fig. 4 A, B).

**Fig. 5:**
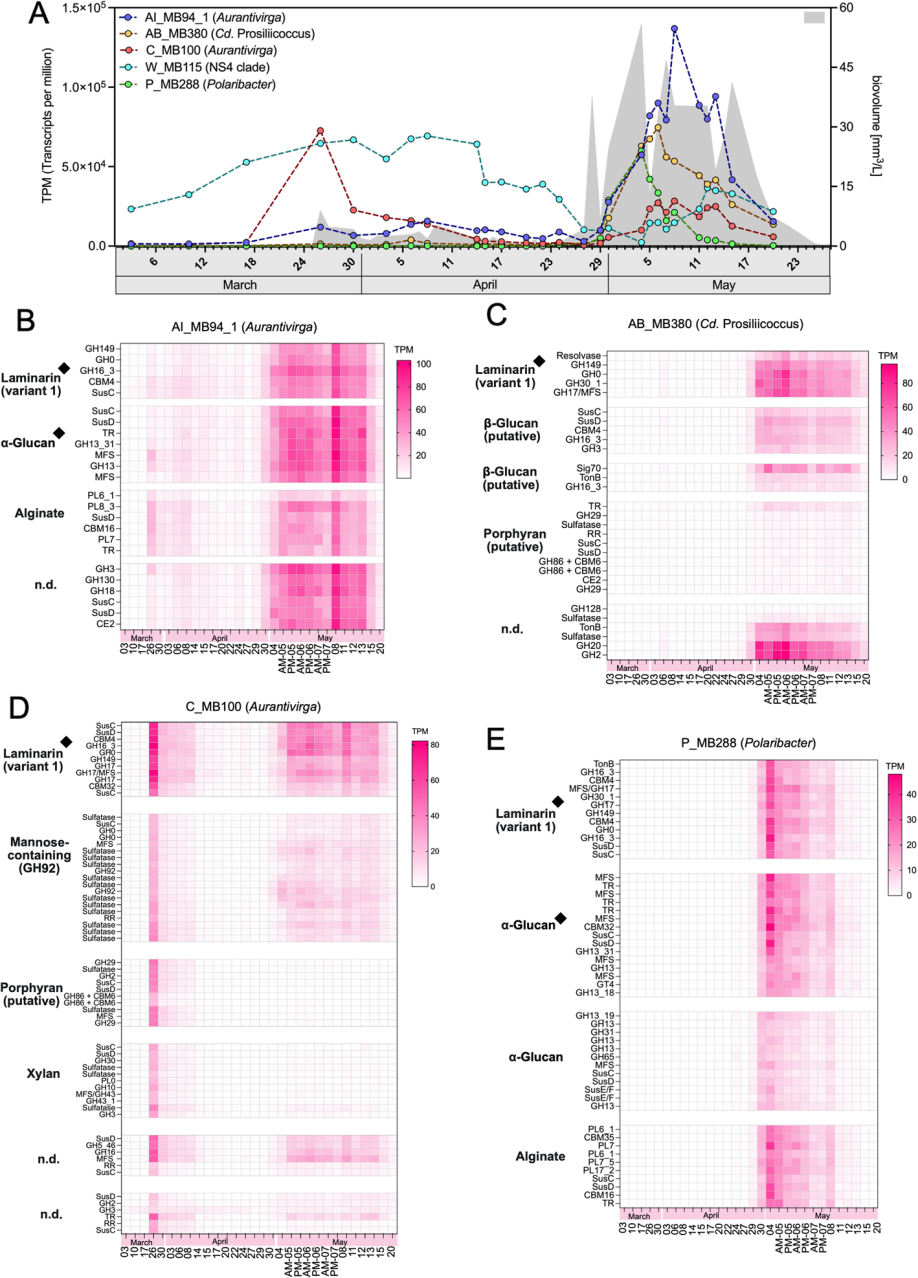
**A** Expression of the five most expressed bacteroidetal MAGs in transcripts per million (TPM) across all sampling time points and total algae biovolumes (mm^3^/L) in the background (turquoise area). **B–E** Corresponding transcriptional profiles of PULs and their predicted polysaccharide substrates. **B** *Aurantivirga* AI_MB94_1. **C** *Cd*. Prosiliicoccus AB_MB380. **D** *Aurantivirga* C_MB100. **E** *Polaribacter* P_MB288. Top expressed laminarin and *α*-glucan PULs are marked (black diamonds)

The five bacteroidetal MAGs with the highest expression were, in descending order, *Aurantivirga* AI_MB94_1, *Cd.* Prosiliicoccus AB_MB380, *Aurantivirga* C_MB100, NS4 clade W_MB115, and *Polaribacter* P_MB288 (Fig. 5A). *Aurantivirga* AI_MB94_1 featured four PULs predicted to target laminarin (CAZymes: GH149-GH0-GH16_3), *α*-glucans (GH13), alginate (PL6-PL8-PL7), and an unspecified polysaccharide (GH3-GH130-GH18-CE2). GH0, GH16_3, and GH13 gene expression levels ranged among the top 10% in this MAG, suggesting particular active laminarin and *α*-glucan degradation (Fig. 5B).

*Cd.* Prosiliicoccus MAG AB_MB380 featured three predicted expressed laminarin PULs (GH149-GH0-GH30_1-GH17, GH16_3-GH3, GH16_3). The first belonged to the previously defined variant 1 type laminarin PULs [48, 73] and exhibited the highest overall expression of these three (Fig. 5C). A fourth PUL, predicted to target porphyran, was not expressed. The second *Cd.* Prosiliicoccus MAG among the topmost expressed MAGs, Q_MB57, had a much weaker overall expression and featured expressed variants 1 and 2 type laminarin PULs, with slightly higher expression of the latter (Fig. S7A in Additional file 1). Specialization of *Cd.* Prosiliicoccus on laminarin corroborates previous analyses [25].

*Polaribacter* MAG P_MB288 featured a similar set of expressed PULs as *Aurantivirga* MAG AI_MB94_1, targeting laminarin (GH149-GH0-GH17-GH16_3-GH30_1), *α*-glucans (GH13-GH65-GH31), and alginate (PL6-PL7-PL17). These two MAGs exhibited an almost synchronous expression peak at the onset of the second bloom phase, indicating competition for the same polysaccharide niche, in which *Aurantivirga* AI_MB94_1 notably outcompeted *Polaribacter* P_MB288 (Fig. 5A). Expression of the PULs in these MAGs correlated well with estimated MAG abundances and furthermore did not change relative to each other over time. Both suggest that these PULs were either unregulated or tightly co-regulated (Fig. 5 B, E). The second *Polaribacter* MAG among the topmost expressed MAGs, X_MB188, was weaker expressed and contained expressed PULs predicted to target *α*-glucans and *α*-mannose-containing sulfated polysaccharides. Respective genes were among the topmost expressed genes in this MAG (Fig. S7B in Additional file 1).

*Aurantivirga* MAGs AB_MB169 and C_MB100 were less active than AI_MB94_1, but featured more diverse expressed PULs predicted to target not only laminarin and *α*-glucans but also xylan (GH43_1-GH10), a putative porphyran (GH29-GH86), a sulfated *α*-mannose-containing polysaccharide (GH92-sulfatases), and additional unspecified glycans (GH2-GH3; GH5-GH16) (Fig. 5; Fig. S6A in Additional file 1). Only the PULs targeting laminarin (variant 1) and the sulfated *α*-mannose-containing polysaccharide (present only in C_MB100) were notably expressed, with all glycoside hydrolases among the top 10% of expressed genes in their respective MAGs. Laminarin-targeting *Aurantivirga* C_MB100 peaked in the first bloom phase, while the *Aurantivirga* Al_MB94_1 peaked in the second bloom with highest expression of its *α*-glucan-targeting PUL (Fig. 5 A, B), providing a prime example of polysaccharide niche distinction within a genus and thereby indicating compositional changes in polysaccharide substrate availability over time.

The *Aurantivirga* MAG with the least overall expression, C_MB344, had only two expressed PULs targeting laminarin and *α*-glucans. In contrast to the PUL in the other three active *Aurantivirga* MAGs, these PULs did not exhibit similar expression patterns over time. While its laminarin PUL was expressed during both bloom phases, its *α*-glucan PUL was only expressed during the first bloom phase. This PUL featured an expressed transcriptional regulator, fortifying the view that this PUL’s expression was downregulated during the second bloom phase (Fig. S6B in Additional file 1).

Additional flavobacterial MAGs affiliating with the NS4 marine group and *Cd.* Abditibacter featured variant 1 laminarin PULs (Figs. S8, S9 in Additional file 1). This PUL in the NS4 clade MAG W_MB115 lacked the characteristic *susCD* gene pair. Further expressed PULs are discussed in the Additional file 1.

**Fig. 6:**
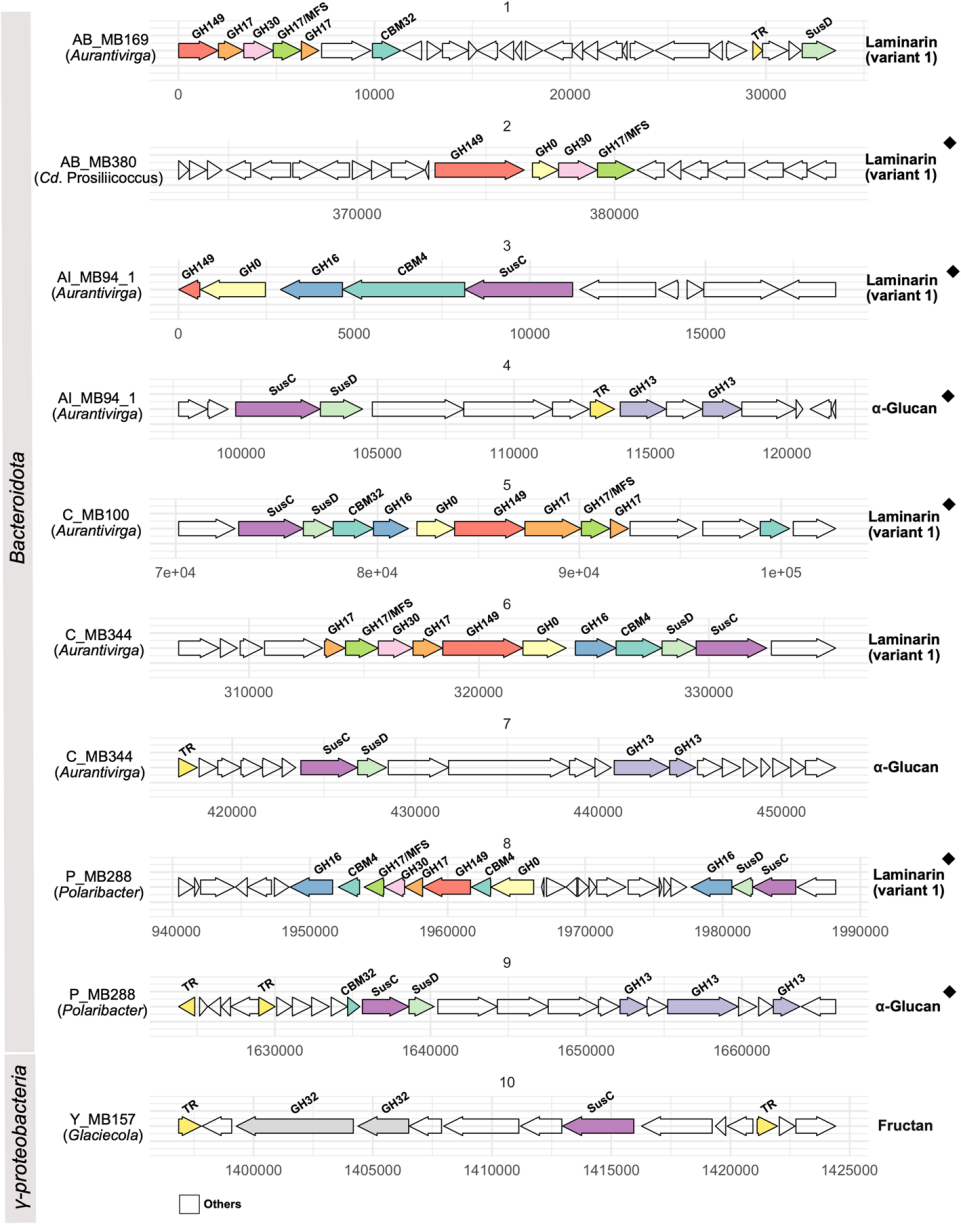
Genetic organization of highly expressed PULs predicted to target different glycans in bacteroidetal and gammaproteobacterial MAGs. PULs from top expressed MAGs mentioned in Fig. 5 are marked (black diamonds)

Variant 1 laminarin PULs were expressed by almost three-quarter (11/15) of the active bacteroidetal and a third (7/18) of the active gammaproteobacterial MAGs (Fig. 3, Fig. 6). Most had at least one fusion of a GH17 with an MFS transporter gene, something that was reported previously for the *Formosa* genus [73]. Such PULs included four of six highly expressed gammaproteobacterial SAR92 MAGs (AK_MB88, B_MB221, V_MB374, X_MB111) (Fig. S10 in Additional file 1), both *Luminiphilus* MAGs (AA_MB219, T_MB10) and one SAR86 (P_MB137_1) MAG, sometimes with an additional GH158 (Figs. S10, S11 in Additional file 1). Unlike bacteroidetal laminarin PULs, these gammaproteobacterial PULs (all SAR92) were flanked by transcriptional regulators (RNA polymerase sigma factor, ECF subfamily, *sigB*), suggesting regulation.

In contrast to these *Gammaproteobacteria*, *Glaciecola* MAG Y_MB157 possessed seven different expressed PULs, but no variant 1 laminarin PUL. Predicted targets included *α*-glucans (GH13-GH31), laminarin/*β*-glucans (GH16_3; GH3-GH16_3; GH3), alginate (PL17_2; PL6_1), and fructans (GH32) (Fig. S12 in Additional file 1). The sole *Verrucomicrobiota* MAG (B_MB250) exhibited expression of genes coding for *α*-L-fucosidases, arylsulfatases, and GH33 family glycoside hydrolases. Details on the predicted metabolism of *Verrucomicrobiota* MAGs obtained from metagenomes off Helgoland Roads were described recently in a dedicated study [56].

### Abundant storage polysaccharides played a pivotal role in the carbon flux between algal primary production and bacterial mineralization

As expected for a diatom-dominated bloom, analyses of high molecular weight dissolved organic matter (HMWDOM) with an anti-*β*-1,3-glucan antibody (BS-400–2) revealed the continuous presence of laminarin (Fig. 4C). PUL expression data also showed highest expression for predicted laminarin PULs, followed by *α*-glucan PULs, alginate PULs, and PULs targeting mannose-rich sulfated polysaccharides, xylan, and porphyran (Fig. 4B). Based on PUL expression data, laminarin was consumed during both bloom phases, with higher expression during the second bloom phase where algal biovolumes were substantially higher (Fig. 4A). Expression of *α*-glucan PULs was lower on overall, in particular during the first bloom phase, but featured a remarkably steep almost fourfold increase at the onset of the second bloom phase (Fig. 4A), suggesting a pronounced increase in soluble *α*-glucans. This confirmed that despite dominance of diatoms during both bloom phases, the response on the bacterioplankton level was rather distinct.

Antibody-based measurements also suggested an abundance of galactose-containing polysaccharides (Additional file 15), specifically galactosyl residues in rhamnogalacturonan I (LM16) and branched (1,6-galactose) (1 → 4)-β-D-galactan (LM26). Intensity data obtained with these two antibodies correlated particularly well with estimated algal biomass data (Fig. S13 in Additional file 1), but interestingly, this was not reflected in corresponding PUL expression in the most abundant MAGs, suggesting a pronounced preference for less complex glycans among the most active bacterioplankton members.

Measurements of monosaccharide concentrations resulting from acid hydrolyzed HMWDOM polysaccharides confirmed that glucose, the monomer of both laminarin and *α*-glucan storage polysaccharides, was the most abundant monosaccharide in the dissolved fraction during the bloom (Fig. S14). Other abundant monosaccharides comprised, in descending order, fucose (monomer in fucoidan and other fucose-containing polysaccharides), galactose (monomer in galactomannan and pectin side chains), mannose/xylose (monomers in mannans/mannose-containing and xylans/xylose-containing polysaccharides), and glucosamine (monomer in e.g., chitin) (Additional file 11). Further monosaccharides of lesser abundance comprised rhamnose, galactosamine, glucuronic acid, arabinose, and galacturonic acid (Fig. S15 in Additional file 1).

## Discussion

The Helgoland 2020 spring bloom was different from the blooms that we previously analyzed at this location. Algal diversity was remarkably low, and the bloom was split in two distinct phases. The first bloom phase was characterized by notable top-down control by copepods, whereas early phases of previous Helgoland spring blooms during which we analyzed bacterioplankton were largely bottom-up controlled (e.g., [72]). Mild winters, which have become increasingly common during recent decades in the German Bight, entail reduced copepod winter mortalities. Resulting higher copepod numbers in spring lead to an earlier onset of grazing in spring phytoplankton blooms as in the past [77]. The copepod bloom during the 2020 Helgoland spring bloom was accompanied by a notable increase in bacterioplankton total cell counts. This agrees with observations that copepod sloppy feeding and excretion can increase substrate availability to bacteria (e.g., [74]).

During the first bloom phase, algal biovolume was dominated by the large diatom species *D. brightwellii*. Blooming *D. brightwellii* are known to inhibit proliferation of other diatom species by toxic metabolites [63]. This may have been one contributing factor to the dominance of *D. brightwellii* during this phase. Size may have been another factor. *D. brightwellii* cells tend to form large cells, in particular in colder waters (< 15 °C) [41], as present at Helgoland Roads in spring (Fig. S1A). *D. brightwellii* observed at Sylt Roads in the North Sea during the sampling period fell into two size classes of 40:120 µm and 70:150 µm in terms of diameter and lengths in pervalvar axis (Additional file 2). Copepods tend to reject very large diatom cells outside of their preferred size range, in particular when these diatoms had sufficient silicate to build thick frustules [65], as was the case during the first bloom phase (Fig. S1C). Still, a certain amount of grazing in concert with diminishing nutrients likely contributed to the reduction of *D. brightwellii* numbers towards the end of the first bloom phase.

The second bloom phase was dominated by *Cerataulina pelagica* and *Chaetoceros* sp. and led to a rapid depletion of silicate, ammonium, and phosphate (Fig. S1 C, D). It is noteworthy that blooming *C. pelagica* have been reported to cope well with diminished silicate concentrations [4]. Likewise, *Chaetoceros* sp. are able to take up silicate and other nutrients at low concentrations [9] and thus can thrive after blooms of other diatoms or under depleted nutrient conditions. This may have played a factor in maintaining dominance throughout the entire second bloom phase, even after nutrients became sparse.

Besides food web effects, also hydrodynamics at Helgoland must be considered, as influxes from distinct water bodies can influence population sizes of planktonic species. Influxes of nutrient-rich, coastal waters were detected at the beginning of the first bloom phase and in particular immediately preceding the second bloom phase. The first influx probably prolonged the first bloom phase, and the second was likely instrumental in the development of the second bloom phase. Neither physicochemical nor bacterioplankton data did suggest a fundamental change in water regime by these influx events. Nevertheless, we cannot exclude that these influxes had quantifiable effects on plankton population sizes, which precludes speculations on causes for population changes during these two well-defined events.

The unusually low algal diversity during both bloom phases allowed testing of whether blooming bacterioplankton clades responded to specific algae. Many bacterioplankton clades correlated well with distinct diatom species. For example, during the initial *D. brightwellii* peak on March 26, bacterioplankton clades such as *Aurantivirga* (C_MB100, C_MB344), NS4, NS2b, *Amylibacter*, and SAR92 (B_MB221) showed sharp expression peaks. Likewise, *Aurantivirga* (AI_MB94_1, AB_MB169), *Polaribacter*, *Cd.* Prosiliicoccus (formerly classified as *Ulvibacter*)*﻿, Glaciecola*, SAR92 (except B_MB221), OM43, *Ilumatobacter*, and *Cd*. Nanopelagicales activities seemed tightly coupled to the presence of *C. pelagica* and *Chaetoceros* sp. diatoms in the second bloom phase. However, in case of the most abundant polysaccharide-degrading bacteria, the presumption of direct coupling to algae species is misleading. Laminarin PUL expression correlated well with overall diatom biomass throughout the bloom, reflecting that diatoms produce laminarin, independent of species. Likewise, the sharp increase in *α*-glucan PUL expression at the onset of the second phase may not have resulted from a change in diatom species composition. During this time, a steep rise in flagellate numbers was accompanied by an about threefold drop in bacterial cell counts from 1.07 × 10^6^ per mL on April 26 down to 3.41 × 10^5^ per mL on April 30 (Fig. 1C). We hypothesize that bacteria die-off, either due to flagellate grazing or other possible means of increased mortality (e.g., viral lysis), led to the release of copious amounts of bacterial storage *α*-glucans that allowed specialized, rivaling clades such as *Aurantivirga* AI_MB94_1 and *Polaribacter* P_MB288 to thrive. In any case, these results demonstrate that shifts in polysaccharide substrate availability can profoundly shape bacterioplankton community composition during bloom events.

During the terminal bloom phase, expressions of all SAR86, *Pelagibacter* (AB_MB223, AI_MB285), OM43 (AB_MB181_1), SAR92 (B_MB221), NS4 (W_MB115), NS2b (AK_MB314_1), and UBA952 (O_MB51_1) MAGs increased. Gammaproteobacterial OM43 clade methylotrophs are frequently associated with algal blooms. They thrive on methanol and other C1 compounds that are released by algae [32]. Likewise, the gammaproteobacterial SAR92 and SAR86 clades are often associated with spring blooms at Helgoland [72]. Some of the abundant clades during the terminal bloom phase are copiotrophs in the sense that they quickly acquire and utilize various low-molecular-weight organic algal substrates. Others, like *Pelagibacterales*/SAR11, are typical oligotrophs that nonetheless can profit from an overall increased availability of nutrients (see [37] for a discussion of the concepts of oligotrophs vs. copiotrophs). As in this study, we have also observed before that absolute SAR11 numbers can increase during spring blooms even though their relative numbers decline (e.g., [72]). Moreover, relative abundances of those *Bacteroidota* that dominated the second bloom phase decreased notably, indicating that they were outcompeted by fast-growing *Gammaproteobacteria* upon the massive release of readily metabolizable algal substrates during the bloom’s short-lived terminal phase.

We have described the diverse CAZyme and PUL repertoires of phytoplankton-associated bacterioplankton clades in previous studies (e.g., [6, 16, 25, 44, 73, 79]). Similar PUL types were also identified in the 2020 spring bloom MAGs presented here. Metatranscriptome analyses, however, revealed that the bulk of PULs that were expressed by the most active bacterioplankton clades targeted only few substrate classes, substantiating hypotheses from previous genome- [44] and metagenome-based studies [48]. These substrates are, from higher to lower expression, laminarin/*β*-glucans, *α*-glucans, alginates, and mannose- and xylose-containing polysaccharides. In particular, variant 1 laminarin PULs, as previously described in *Formosa* sp. Hel1_33_131 [73], exhibited high expression levels. High expression of similar laminarin PULs in 18 abundant MAGs (Figs. 5, 6 and Figs. S6–11 in Additional file 1) suggested a stiff competition for laminarin among dominant bacterioplankton clades.

We observed distinct polysaccharide preferences not only among members of distinct clades, but also within abundant members of the same genus. For instance, we found high expression of GH92 *α*-mannosidases in *Aurantivirga* C_MB100 as well as expressed laminarin and xylan PULs, whereas *Aurantivirga* AI_MB94_1 expressed laminarin, *α*-glucan, and alginate PULs. These preferences seemed to be either hardcoded or tightly co-regulated, because overall PUL expression patterns did not change in most *Aurantivirga* MAGs. An exception was *Aurantivirga* MAG C_MB344 that showed a notable downregulation of its *α*-glucan PUL relative to its laminarin PUL during the second bloom phase (Fig. S6B in Additional file 1).

We have shown before that heterotrophic bacterioplankton clades that respond rapidly to phytoplankton blooms often have relatively small genomes of around 2 Mbp and only few PULs [48]. The top 50 expressed MAGs in this study mostly had genome sizes between 1 Mbp (*Pelagibacter* and OM43 clade) and 3 Mbp, except *Luminiphilus* T_MB10, OM182 P_MB234_1, and *Ilumatobacter* C_MB28, whose MAGs ranged up to ~ 3.5 Mbp. The highest expressed MAG during the second bloom phase, *Aurantivirga* AI_MB94_1 and *Cd.* Prosiliicoccus AB_MB380, had sizes around 2.0 to 2.2 Mbp. Regulation is among those traits that are typically reduced during genome streamlining — *Pelagibacter ubique* being a prime example [27]. Having largely unregulated PULs without obvious transcriptional regulators (i.e., without genes with identifiable regulator motifs) could be an adaptation of *Bacteroidota* for a swift response to bloom situations and might be among the reasons why *Bacteroidota* usually outcompete *Gammaproteobacteria* at the onset of spring phytoplankton blooms at Helgoland Roads (e.g., [71, 72]).

During the last decade, we have isolated thousands of North Sea bacterial strains and classified them using 16S rRNA gene sequencing [1, 30, 31]. Based on comparisons with in situ 16S rRNA amplicon and metagenome data, we found only two flavobacterial *Formosa* strains [73] and one gammaproteobacterial *Reinekea* strain [5, 30] that were almost identical to abundant bacterioplankton species during blooms (as evidenced by full genome sequencing). In addition, we could reconstruct the full genome of flavobacterial *Cd. *Prosiliicoccus vernus from bloom-associated metagenome data [25]. Still, most of the actual bloom-associated planktonic bacteria remain uncultivated, which is why high-quality MAGs together with in situ expression data represent invaluable sources of information on the actual ecophysiological roles of abundant bloom-associated bacteria.

### Concluding remarks

Microbial decomposition of algal biomass is a concerted effort of both bacterial specialists and generalists with distinct substrate niches. While resource partitioning is particularly evident for polysaccharides (e.g., [44]), it also plays a role for many other abundant substrates, which are beyond the focus of this particular study. Availability and composition of algae-derived substrates therefore exert a strong influence in shaping the bacterioplankton community during phytoplankton blooms. As we show in this study, abundant, soluble, and structurally simple storage glycans, such as laminarin and *α*-glucans, play a pivotal role in this process. Also, alginates seem to be readily metabolized, which not only constitute a cell wall component of brown algae but are also produced by some bloom-associated bacteria [26]. However, little did we find about the fate of more structurally complex polysaccharides. These might be preferentially metabolized by particle-associated bacteria or, in case these particles sink, by bacteria in the upper sediment. While planktonic bacteria constitute the bulk of bacteria during algal blooms, future studies should therefore include particle-associated bacteria to ultimately gain a more quantitative understanding about polysaccharide-based carbon fluxes during phytoplankton bloom events.

## Materials and methods

### Sampling and filtration

Samples were taken from March to May 2020 at the long-term ecological research site “Kabeltonne” (54° 11.3′ N, 7° 54.0′ E) off the coast of Helgoland Roads (North Sea) during a diatom-dominated spring phytoplankton bloom. Seawater was sampled at 1 m depth five times a week as described previously [72] for a total of 55 days.

Ten liters of seawater were filtered sequentially through 10, 3, and 0.2 µm pore-size polycarbonate filters (142 mm diameter) for bacterial biomass. The 10 and 3 µm filters retained most of the eukaryotes, larger particles, and attached bacteria, while the bulk of the free-living bacteria were collected on the 0.2 µm filters. All filtrations were performed in duplicates, and filters were immediately flash frozen in liquid nitrogen and kept at − 80 °C until further use.

For cell counting, 10 or 100 mL of unfiltered seawater was fixed with 37% formaldehyde (v/v) to a concentration of 1% (v/v) for 1 h at room temperature. The fixed samples were then filtered directly onto 0.2 µm pore-sized polycarbonate filters (47 mm diameter). All filters were preserved at − 80 °C until further use.

### Cell counts of total bacteria and of specific clades

Bacterial cells were stained with DAPI (4′,6-diamidino-2-phenylindole) and counted microscopically for total cell number estimates. Likewise, cells of prominent clades were stained with specific probes for CARD-FISH and counted microscopically. Both techniques were applied as described previously [72]. CARD-FISH probes were as follows: AUR452 (*Aurantivirga*), POL740 (*Polaribacter*), Vis6-814 (Vis6 clade including *Cd. *Abditibacter), SAR86-1245 (SAR86 clade), SAR92-627 (SAR92 clade), OM182-707 (OM182 clade), SAR11-mix (SAR11 clade including *Pelagibacter*), and Ros537 (*Roseobacter* clade). Corresponding sequences are provided in Additional file 13.

### Physicochemical and phytoplankton measurements

Wind direction data were obtained from the Climate Data Store of the Copernicus Climate Change Service [34]. All other physicochemical parameters, such as temperature, salinity, Secchi depth, nitrate, nitrite, ammonium, phosphate, silicate as well as phytoplankton cell numbers and community composition, were obtained as part of the Helgoland Roads time series [47, 78]. Phytoplankton biovolumes were determined according to Hillebrandt et al. 1999 [35] in the framework of the Sylt Roads time series (see [3] for details). Both the Helgoland and Sylt Roads time series are conducted by the Alfred Wegener Institute, Helmholtz Centre for Polar and Marine Research (Bremerhaven, Germany).

### Sequencing

Metagenome and metatranscriptome sequencing of biomass from the 0.2 µm filters were performed at the Max Planck Genome Centre (Cologne, Germany). For each time point, one filter was used for the extraction of both DNA and RNA. Isolated RNA was processed to deplete ribosomal RNAs using oligo-based customized probes [54], and the remaining RNA was further used for sequencing. On the basis of chlorophyll *a* data and cell numbers, we selected 27 time points for sequencing. On three dates, samples were taken twice a day (8 AM, 9 PM), in order to possibly test expression with and without sunlight (not part of this study). This amounts to a total of 30 metagenomes and corresponding metatranscriptomes. Sampling dates and further details are provided in Additional file 14.

### Metagenomics

#### Sequencing, assemblies, and binning

Metagenome sequencing was performed on a PacBio Sequel II (Pacific Biosciences, Menlo Park, CA, USA) using one SMRT cell per sample in long-read HiFi mode. Details on the raw data are provided in Additional file 14. Assemblies were generated using Flye v2.8.3 [45] with options − *meta* and − *pacbio-hifi*. 16S rRNA sequences were extracted from metagenome assemblies using Barrnap v0.9 [68] and classified using SILVAngs and the Silva database v138.1 [60, 61]. A similarity threshold of 0.97 was used to cluster the sequences for creating OTUs (operational taxonomic units).

Reconstruction of MAGs (metagenome-assembled genomes) was carried out in Anvi’o v7.0 [20]. Initial bins were created with metabat2 [43] from within Anvi’o, refined by invoking the anvi-refine command, and subsequently inspected visually in terms of GC profiles and contig coverages. CheckM v1.0.18 [57] was used to assess MAG completeness and contamination. Since assembly and binning were performed on a per sample basis, redundant MAGs were obtained. We used dRep v3.2.0 [55] to dereplicate MAGs with > 70% completeness and < 5% contamination at 0.95 ANI (average nucleotide identity). MAG phylogenomic affiliations were assigned using GTDB-Tk v1.3.0 [14] with GTDB v202. The taxonomic affiliation of representative MAGs was further improved by extracting 16S rRNA sequences from the MAGs and placing them in the tree using Arb v7.0 [52] and Silva v138.1.

### Metatranscriptomics

#### RNA read quality filtering and mapping

Metatranscriptome sequencing was performed on an Illumina HiSeq 3000 (Illumina Inc., San Diego, CA, USA). About 100 million paired-end reads (2 × 150 bp) were generated per sample. Ribosomal RNA reads were filtered using SortMeRNA v3.0 [46]. Remaining messenger RNA reads were quality trimmed and end repaired using the *bbduk* and *repair* scripts of the BBMap v35.14 suite (https://sourceforge.net/projects/bbmap/). Reads with a minimum read length of 70 bp were subsequently mapped onto all 251 representative MAGs using Bowtie2 [49] as a part of the SqueezeMeta v1.3.1 pipeline [70]. Mapping statistics are provided in Additional file 14.

### Integrated analysis

We used SqueezeMeta in merged mode for the integrated analysis of metagenomes and corresponding metatranscriptomes. Concatenated MAGs were supplied to SqueezeMeta as metagenome assembly. Contig statistics were calculated using Prinseq v0.20.4 [66]. RNAs were predicted using Barrnap and subsequently classified using the RDP classifier [76]. Aragorn [50] was used for the prediction of tRNA/tmRNA sequences. ORFs were predicted externally using FragGeneScan (parameters *w1* and *sanger_5*) [62] and searched against GenBank r239 [17], eggNOG v5.0 [38], KEGG r58.0 [42], and CAZy (as of 30 July 2020) [12] using Diamond [11]. HMM homology searches were done using HMMER3 [18] against the Pfam 33.0 database [24]. Combined annotations were used for manual prediction of PULs and CAZyme clusters. We denominated co-localizations of CAZyme, s*usCD*, or s*usC* genes as PULs and co-localizations of CAZyme genes without *susCD* or with other transporters (e.g., of the MFS type) as CAZyme clusters. Mapping of mRNA reads against contigs (concatenated MAGs) was performed using Bowtie2, and transcripts per million (TPM) values were calculated for all MAGs of a given sample as follows: (Σ reads of sample successfully mapping to a MAG × 10^6)/(Σ lengths of contigs of the MAG × Σ number of reads in the sample). Results were visualized using the SQMtools R package.

### Saccharide measurements

High molecular weight DOM (HMWDOM) was sampled using tangential flow filtration in parallel to OMICs sampling from the same water body. Samples were processed as described previously [75] with slight modifications (see Additional file 1). In brief, polysaccharides from HMWDOM samples were extracted and analyzed using carbohydrate microarrays in combination with monoclonal antibodies specific for various polysaccharides (Additional file 15). Antibody-binding signal intensities were quantified with Array-Pro Analyzer 6.3 (Media Cybernetics Inc., Rockville, MD, USA).

Aliquots of the HMWDOM samples were also used for monosaccharide analysis by high-performance anion-exchange chromatography with pulsed amperometric detection (HPAEC-PAD) with a Dionex CarboPac PA10 column (ThermoFisher Scientific, Waltham, MA, USA) as described elsewhere [19, 75].

## Supplementary Information

**Additional file 1:** **Fig. S1.** A. Levels of salinity and temperature. B. Total bacterial cell counts and flagellate counts. C, D. Concentrations of  silicate, phosphate, nitrate, and ammonium. **Fig. S2.** Wind directions and speeds at 10 m above the sea surface level as obtained from the Climate Data Store of the Copernicus Climate Change Service (ERA 5 product). Corresponding wind vector data is provided in Additional file 4. **Fig. S3.** Quality measures of the MAGs obtained in this study. **Fig. S4.** Overall expression of all mentioned clades in terms of transcripts per million during the 2020 North Sea spring bloom. **Fig. S5.** Transcription pattern of seven archaeal MAGs obtained in this study. **Fig. S6.** Transcriptional profiles of PULs and their predicted polysaccharide substrates in *Aurantivirga* MAG AB_MB169 and C_MB344. **Fig. S7.** Transcriptional profiles of PULs and their predicted polysaccharide substrates in *Cd. *Prosiliicoccus MAG Q_MB57 and *Polaribacter *MAG X_MB288. **Fig. S8.** Transcriptional profiles of PULs and their predicted polysaccharide substrates in *Abditibacter *MAG D_MB74 and L_MB280. **Fig. S9.** Transcriptional profiles of PULs and their predicted polysaccharide substrates in NS4 clade MAG W_MB115. **Fig. S10.** Transcriptional profiles of PULs and their predicted polysaccharide substrates in all SAR92 clade MAGs: AK_MB88_1, B_MB221, X_MB111, V_MB374, AB_MB236 and SAR86 MAG: P_MB137_1. **Fig. S11.** Transcriptional profiles of PULs and their predicted polysaccharide substrates in *Luminiphilus* OM60 (NOR5) clade MAGs: AA_MB219 and T_MB10. **Fig. S12.** Transcriptional profiles of PULs and their predicted polysaccharide substrates in the highly expressed and sole *Glaciecola *MAG Y_MB157. **Fig. S13.** Antibody-based measurements (LM16, specific for galactosyl residues in rhamnogalacturonan I and LM26, specific for branched (1,6-galactose)(1→4)-β-D-galactan) of galactose-containing polysaccharides extracted either with MilliQ or EDTA (lines) as compared to the estimated algal biomass data (gray area). **Fig. S14.** Comparison of the combined expression of PULs targeting laminarin and α-glucan storage polysaccharides that both consist entirely of glucose, and the measured concentrations of glucose in polysaccharides from HMWDOM. **Fig. S15.** Measured concentrations of monosaccharides other than glucose in HMWDOM across samples.

**Additional file 2:** Total algal cell counts and biovolumes (BV), inorganic nutrients and precipitation measured during the 2020 Helgoland spring phytoplankton bloom.

**Additional file 3:** Copepod counts during the 2020 Helgoland spring phytoplankton bloom.

**Additional file 4:** Wind vector components, direction and speed data at 10 m above the sea surface. 

**Additional file 5:** Sampling dates and statistics of 16S rRNA sequences extracted from PacBio Sequel II raw reads.

**Additional file 6:** Relative abundances of 16S rRNA gene sequences extracted from metagenomes.

**Additional file 7:** Microscopic cell counts with fluorescently labeled CARD-FISH probes.

**Additional file 8:** Numbers of bins obtained by metagenome binning using Anvi'o.

**Additional file 9:** GTDB affiliation of all MAGs dereplicated at 0.95 ANI along with other genomic features.

**Additional file 10:** TPM (transcripts per million) values of major clades during the 2020 Helgoland spring phytoplankton bloom.

**Additional file 11:** Monosaccharide concentrations resulting from acid hydrolysis of high molecular weight DOM polysaccharides.

**Additioanl file 12:** Antibody-based (BS-400-2, specific for β-1,3-glucan) measurements of dissolved laminarin extracted either with MilliQ or EDTA.

**Additional file 13:** CARD-FISH probes used in this study.

**Additional file 14:** Sampling dates and statistics of raw data obtained for metagenomes and metatranscriptomes.

**Additional file 15:** Polysaccharide measurements during the 2020 Helgoland Spring phytoplankton bloom.

## Data Availability

Metagenome, metatranscriptome, and MAG sequence data are available from the European Nucleotide Archive (accession PRJEB52999). Supporting environmental data is also available as Zenodo repository (https://doi.org/10.5281/zenodo.7656261).
