# Supplementary material for: Dissolved storage glycans shaped the community composition of abundant bacterioplankton clades during a North Sea spring phytoplankton bloom
Source: Microbiome. 2023 Apr 17;11:77. doi: 10.1186/s40168-023-01517-x (PMC10108472; doi:10.1186/s40168-023-01517-x)
Supplement: Supplementary file 2 — Additional file 1: Fig. S1. A. Levels of salinity and temperature. B. Total bacterial cell counts and flagellate counts. C, D. Concentrations of silicate, phosphate, nitrate, and ammonium. Fig. S2. Wind directions and speeds at 10 m above the sea surface level as obtained from the Climate Data Store of the Copernicus Climate Change Service (ERA 5 product). Corresponding wind vector data is provided in Additional file 4. Fig. S3. Quality measures of the MAGs obtained in this study. Fig. S4. Overall expression of all mentioned clades in terms of transcripts per million during the 2020 North Sea spring bloom. Fig. S5. Transcription pattern of seven archaeal MAGs obtained in this study. Fig. S6. Transcriptional profiles of PULs and their predicted polysaccharide substrates in Aurantivirga MAG AB_MB169 and C_MB344. Fig. S7. Transcriptional profiles of PULs and their predicted polysaccharide substrates in Cd. Prosiliicoccus MAG Q_MB57 and Polaribacter MAG X_MB288. Fig. S8. Transcriptional profiles of PULs and their predicted polysaccharide substrates in Abditibacter MAG D_MB74 and L_MB280. Fig. S9. Transcriptional profiles of PULs and their predicted polysaccharide substrates in NS4 clade MAG W_MB115. Fig. S10. Transcriptional profiles of PULs and their predicted polysaccharide substrates in all SAR92 clade MAGs: AK_MB88_1, B_MB221, X_MB111, V_MB374, AB_MB236 and SAR86 MAG: P_MB137_1. Fig. S11. Transcriptional profiles of PULs and their predicted polysaccharide substrates in Luminiphilus OM60 (NOR5) clade MAGs: AA_MB219 and T_MB10. Fig. S12. Transcriptional profiles of PULs and their predicted polysaccharide substrates in the highly expressed and sole Glaciecola MAG Y_MB157. Fig. S13. Antibody-based measurements (LM16, specific for galactosyl residues in rhamnogalacturonan I and LM26, specific for branched (1,6-galactose)(1→4)-β-D-galactan) of galactose-containing polysaccharides extracted either with MilliQ or EDTA (lines) as compared to the estimated algal biomass data [file 40168_2023_1517_MOESM1_ESM.docx]

**Additional File 1**

*Dissolved storage glycans shaped the community composition of abundant bacterioplankton clades during a North Sea spring phytoplankton bloom*

Chandni Sidhu^1^, Inga V. Kirstein^2^, Cédric L. Meunier^2^, Johannes Rick^3^, Vera Fofonova^4^, Karen H. Wiltshire^2^, Nicola Steinke^1,5^, Silvia Vidal-Melgosa^1,5^, Jan-Hendrik Hehemann^1,5^, Bruno Huettel^6^, Thomas Schweder^7,8^, Bernhard M. Fuchs^1^*, Rudolf I. Amann^1^*, Hanno Teeling^1^*

^1^ Max Planck Institute for Marine Microbiology, Celsiusstraße 1, 28359 Bremen, Germany

^2^ Alfred Wegener Institute for Polar and Marine Research, Biologische Anstalt Helgoland, P.O. Box 180, 27483 Helgoland, Germany

^3^ Alfred Wegener Institute for Polar and Marine Research, Hafenstraße 43, 25992, List/Sylt, Germany

^4^ Alfred Wegener Institute for Polar and Marine Research, Klußmannstraße 3, 27570, Bremerhaven, Germany

^5^ Center for Marine Environmental Sciences, MARUM, University of Bremen, Leobener Straße 8, 28359 Bremen, Germany

^6^ Max Planck Genome Centre Cologne, Carl von Linné-Weg 10, 50829 Köln, Germany

^7^ Institute of Pharmacy, University of Greifswald, Felix-Hausdorff-Straße 3, 17489 Greifswald, Germany

^8^ Institute of Marine Biotechnology, Walther-Rathenau-Straße 49a, 17489 Greifswald, Germany

^*^ Corresponding authors:

Hanno Teeling, Max Planck Institute for Marine Microbiology, Celsiusstraße 1, 28359 Bremen, e-mail: hteeling@mpi-bremen.de, phone: +49 421 2028 9760

Rudolf I. Amann, Max Planck Institute for Marine Microbiology, Celsiusstraße 1, 28359 Bremen, e-mail: ramann@mpi-bremen.de, phone: +49 421 2028 9300

Bernhard M. Fuchs, Max Planck Institute for Marine Microbiology, Celsiusstraße 1, 28359 Bremen, e-mail: [bfuchs@mpi-bremen.de](mailto:bfuchs@mpi-bremen.de), phone: +49 421 2028 9350

E-mail addresses and telephone numbers of all authors:

Chandni Sidhu [csidhu@mpi-bremen.de](mailto:csidhu@mpi-bremen.de) +49 421 2028 9582

Inga V. Kirstein [inga.kirstein@awi.de](mailto:inga.kirstein@awi.de) +49 4725 819 3153

Cédric L. Meunier [Cedric.Meunier@awi.de](mailto:Cedric.Meunier@awi.de) +49 4725 819 3143

Johannes Rick [johannes.rick@awi.de](mailto:johannes.rick@awi.de) +49 4651 956 4220

Vera Fofonova [Vera.Fofonova@awi.de](mailto:Vera.Fofonova@awi.de) +49 471 4831 1722

Karen H. Wiltshire Karen.Wiltshire@awi.de +49 4651 956 4112

Nicola Steinke [nsteinke@mpi-bremen.de](mailto:nsteinke@mpi-bremen.de) +49 421 2028 7360

Silvia Vidal-Melgosa [svidal@mpi-bremen.de](mailto:svidal@mpi-bremen.de) +49 421 2028 7360

Jan-Hendrik Hehemann [jheheman@mpi-bremen.de](mailto:jheheman@mpi-bremen.de) +49 421 2028 7360

Bruno Huettel huettel@mpipz.mpg.de +49 221 5062 828

Thomas Schweder schweder@uni-greifswald.de +49 3834 420 4212

Bernhard M. Fuchs bfuchs@mpi-bremen.de +49 421 2028 9350

Rudolf I. Amann ramann@mpi-bremen.de +49 421 2028 9300

Hanno Teeling hteeling@mpi-bremen.de +49 421 2028 9760

**Conflict of interest**

The authors declare no conflict of interest.

**Additional Results**

*Additional highly expressed MAGs*

In the following section we describe MAGs that belonged to the 50 topmost expressed MAGs, but were not described in the main manuscript:

*- Actinobacteriota*

Three out of ten *Actinobacteriota* MAGs (AA_MB45, C_MB28, and C_MB123) were present among the topmost 50. MAG AA_MB45 affiliated with the *Actinomarina* genus and exhibited highest expression during the first bloom phase, whereas the other two affiliating with *Ilumatobacter* (C_MB28) and *Cd*. Nanopelagicales (C_MB123) exhibited their highest expression during the late bloom.

*- Verrucomicrobiota*

Only one out of eleven *Verrucomicrobiota* MAGs belonged to the 50 topmost expressed MAGs. This MAG (B_MB250) affiliated with the BACL24 clade (*Opitutaceae*) and showed comparatively low expression levels during the pre-bloom and first bloom phases (**Fig. 3**).

*- Archaea*

The top 50 expressed MAGs also contained three archaeal MAGs, all of which belonged to the *Poseidoniaceae* family (class *Poseidoniia*). Expression of these MAGs was limited to the pre-bloom period and reached its maximum TPM on March 26^th^. One of these MAGs (W_MB9_1) contributed no less than 121,680 TPM (12.2%) on this date. The other two (AF_MB43, AG_MB45) also attained maximum expression on March 26^th^, contributing 38,571 (3.9%) and 25,843 (2.6%) TPM, respectively (Fig. 3B). Only minor expression was detected afterwards, indicating a negligible role of *Archaea* during the bloom (**Fig. S5**).

*- Gammaproteobacteria*

A single MAG (A_MB1) affiliating with *Thioglobus* A (SUP05 clade) exhibited highest expression before the first bloom peak on March 26^th^, suggesting that this thiotrophic clade did not benefit from the algal bloom in the same way as other *Gammaproteobacteria*. Finally, a single MAG (P_MB234_1) affiliating with OM182 was present among the top 50 expressed MAGs, and was expressed throughout all bloom phases to varying degrees.

*Polysaccharide degradation genes present in other MAGs*

In *Bacteroidota* the NS2b clade MAG AK-MB314_1 lacked laminarin and α-glucan PULs, but did have PULs possibly targeting sialic acids (GH33-GH3), with GH3 among the topmost expressed genes. This MAG also featured highly expressed GH92 (α-1,2-mannosidase) genes arranged in a PUL-like structure along with *susCD* genes. In the NS3a clade MAG F_MB366, a GH13 of a putative α-glucan PUL was among the top 10% expressed genes. Finally, MAG O_MB51_1 of the *Crocinitomicaceae-*affiliating UBA952 clade featured high expression of a GH16-containing laminarin PUL during the terminal bloom phase (data not shown).

Similar to many *Bacteroidota*, *Glaciecola* MAG Y_MB157 featured high expression of TonB-dependent receptors as well as expression of additional CAZymes, such as a CE4, GH13 and sugar kinase genes among the top 10% of its expressed genes.

*Monosaccharide composition of DOM*

Measurements of monosaccharide concentrations resulting from acid hydrolyzed high molecular weight DOM polysaccharides confirmed that glucose, the monomer of both laminarin and α-glucan storage polysaccharides, was the most abundant monosaccharide in the dissolved fraction during the bloom (**Fig. S14)**. Other abundant monosaccharides comprised, in descending order, fucose (monomer in fucoidan and other fucose-containing polysaccharides), galactose (monomer in galactomannan and pectin side chains), mannose/xylose (monomers in mannans/mannose-containing and xylans/xylose-containing polysaccharides), and glucosamine (monomer in e.g. chitin) (**Additional file 11**). Further monosaccharides of lesser abundance comprised rhamnose, galactosamine, glucuronic acid, arabinose and galacturonic acid (**Fig. S15**).

**Additional Materials and Methods**

*Sampling and processing of HMWDOM*

High molecular weight dissolved organic matter (HMWDOM) samples were obtained with a procedure described previously (Vidal-Melgosa *et al.*, 2021) with few modifications. In brief, 100 L of 0.2 μm-filtered seawater samples were concentrated to a final volume of 0.5 L using a Sartoflow Study tangential flow filtration (TFF) system (Sartorius Stedim, Göttingen, Germany). The TFF system was run with three filter cassettes with a 10 kDa cutoff (3051463901E--SW, Sartocon® Slice PESU Cassette, 0.1 m² filtration area, Sartorius Stedim). The concentrated samples containing molecules <0.2 μm and >10 kDa were stored at -20 °C until further use. After the sampling campaign, samples were further concentrated using an Amicon stirred ultrafiltration cell (Merck Millipore) with a 1 kDa membrane (76 mm PES, Sartorius), subsequently dialyzed (1 kDa cutoff) and freeze-dried as described previously (Vidal-Melgosa *et al.*, 2021). Sampling dates: 03/03/2020, 05/03/2020, 06/03/2020, 10/03/ 20220, 17/03/2020, 19/03/2020, 24/03/2020, 26/03/2020, 30/03/2020, 01/04/2020, 03/04/2020, 06/04/2020, 08/04/2020, 15/04/2020, 17/04/2020, 20/04/2020, 22/04/2020, 24/04/2020, 27/04/2020, 29/04/2020, 05/05/2020, 06/05/2020, 07/05/2020, 11/05/2020, 13/05/2020, 15/05/2020, 20/05/2020.

*HMWDOM monosaccharide analysis by HPAEC-PAD*

For chemical glycan hydrolysis, 25 μL triplicates of each Amicon-concentrated DOM sample were incubated in 1 M HCl (475 μL Milli‐Q water, 500 μL 2 M HCl) in sealed glass ampoules for 24 h at 100 °C. Afterwards, 800 μL aliquots of hydrolysate were evaporated using a RVC2-18 CD plus HCl-resistant speed‐vac (Martin Christ Gefriertrocknungsanlagen GmbH, Osterode am Harz, Germany) and resuspended in 400 μL Milli‐Q water. This corresponded to a 1:20 dilution of the Amicon-concentrated DOM samples. Monosaccharide standard solutions were prepared in an analogous manner in 1 M HCl, evaporated and resuspended in Milli‐Q water. Monosaccharide contents were determined using High-Performance Anion-Exchange Chromatography with Pulsed Amperometric Detection (HPAEC‐PAD) with a DionexCarboPac PA10 column (Thermo Scientific) as described elsewhere (Engel & Händel, 2011).

*HMWDOM polysaccharide analysis by carbohydrate microarrays and monoclonal antibodies*

Polysaccharide extraction of the HMWDOM samples and carbohydrate microarray analysis with antibodies was performed as described previously (Vidal-Melgosa *et al.*, 2021). In brief, freeze-dried HMWDOM samples were homogenized, and polysaccharides were sequentially extracted with: autoclaved MilliQ water, 300 mM EDTA pH 7.5, and with 4 M NaOH containing 0.1% w/v NaBH4. Polysaccharide extracts were subsequently printed onto 0.45 µm pore-sized nitrocellulose membranes (Whatman, Little Chalfont, Buckinghamshire, UK) using a microarray robot (Sprint, Arrayjet, Roslin, UK). Before printing, all extracts were diluted twofold in printing buffer (55.2% glycerol, 44% water, 0.8% Triton X-100). Due to high viscosities of the MilliQ extracts, these were first diluted ninefold in MilliQ and then diluted twofold in printing buffer. Each sample extract was then printed in four replicates, thus being represented by four spots in the microarray. The arrays were probed with polysaccharide-specific monoclonal antibodies, and those were detected with secondary antibodies conjugated to alkaline phosphatase. Microarrays were developed in a solution containing 5-bromo-4-chloro-3-indolylphosphate and nitroblue tetrazolium in alkaline phosphatase buffer. Afterwards, the color signal intensity was quantified using Array-Pro Analyzer 6.3 (Media Cybernetics Inc., Rockville, MD, USA). The highest detected signal intensity, which was with the antibody JIM13 on the EDTA extract of the 6^th^ of May sample, was set to 100 and all other values were normalized accordingly. The microarray data shown in Fig. 4C correspond to probing with the mouse monoclonal antibody BS-400-2 (RRID: AB_2747399; BioSupplies, Bundoora, Australia) and an anti-mouse secondary antibody conjugated to alkaline phosphatase (A3562, Sigma-Aldrich, St. Louis, MA, USA). These data show the mean antibody signal intensity and error bars denote the standard deviation of the four printing replicates. Further details are provided in Vidal-Melgosa *et al.* (Vidal-Melgosa *et al.*, 2021) and further information on the antibodies including initial results are summarized in **Additional file 15.**

**Additional Discussion**

*Study limitations*

*- CARD-FISH probes*

CARD-FISH probes were chosen based on the abundance of microbial clades in the metagenome dataset. The specificities of these probes depend on the underlying taxonomic framework. For our study we used the taxonomy of the ARB-SILVA project and its corresponding 16S rRNA sequence database. For each probe we ensured that its specificity matched with the taxonomic classification of the 16S rRNA genes from the metagenome dataset. However, for some alphaproteobacterial clades, such as *Amylibacter* and *Planktomarina*, we failed to devise suitable probes. The main reason is the high diversity within these clades that renders probe design almost impossible.

*- Carbohydrate microarrays*

The applied antibody-based microarrays can only detect glycans for which a specific antibody is available. Even though we have a library of >50 antibodies, we cannot rule out that there might have been glycans present during algae blooms at Helgoland Roads for which we did not have suitable antibodies.

The method is semi-quantitative as it does not allow for detection of the absolute concentration of a glycan epitope in a sample. Still it is possible to not only detect presence of a glycan epitope, but also to determine its relative abundance. The relative abundance of an epitope is determined by the signal reported by antibody binding. We have demonstrated the relationship between epitope concentration and antibody signal intensity before, for example in Vidal-Melgosa *et al.* (2021) where several polysaccharides were printed including serial dilutions, which after probing resulted in antibody signals that correlated with epitope concentrations. Here we could detect presence of β-1,3-glucan epitopes and follow changes of its relative abundance over time.

Our microarray approach allows immobilization of polysaccharides and long oligosaccharides, but not of monosaccharides and short oligosaccharides (Pedersen *et al*., 2012; Vidal-Melgosa *et al.*, 2015). Furthermore, we only sampled HMWDOM >10 kDa. Therefore, we did not detect short oligosaccharides. However, this did not result in underestimation of the saccharide content (i.e. detection of less epitope relative signal) of HMWDOM.

*- Bioinformatics and statistics*

MAGs are more likely to reflect consensus core genomes than actual *in situ* genomes with all accessory genes. However, our previous studies have shown that in particular fast responders to phytoplankton blooms often feature an astonishing level of clonality (Avcı *et al.*, 2020), which is why this is unlikely to affect results. Likewise, not all polysaccharide degradation capacities are encoded in PULs, which could lead to an underestimation of bacterial polysaccharide turnover. This might affect in particular simple storage polysaccharides such as laminarin and α-glucans, which require only few genes for degradation. To make this comprehensive study feasible, we forewent sample replication in favor of a high time resolution over a long sampling period. Dense sampling over time compensates for replication to some extent and still allows for meaningful analyses (Lennon, 2011). However, it imposes limits on statistical analyses, which is why we restricted our analyses to the most pronounced major effects.


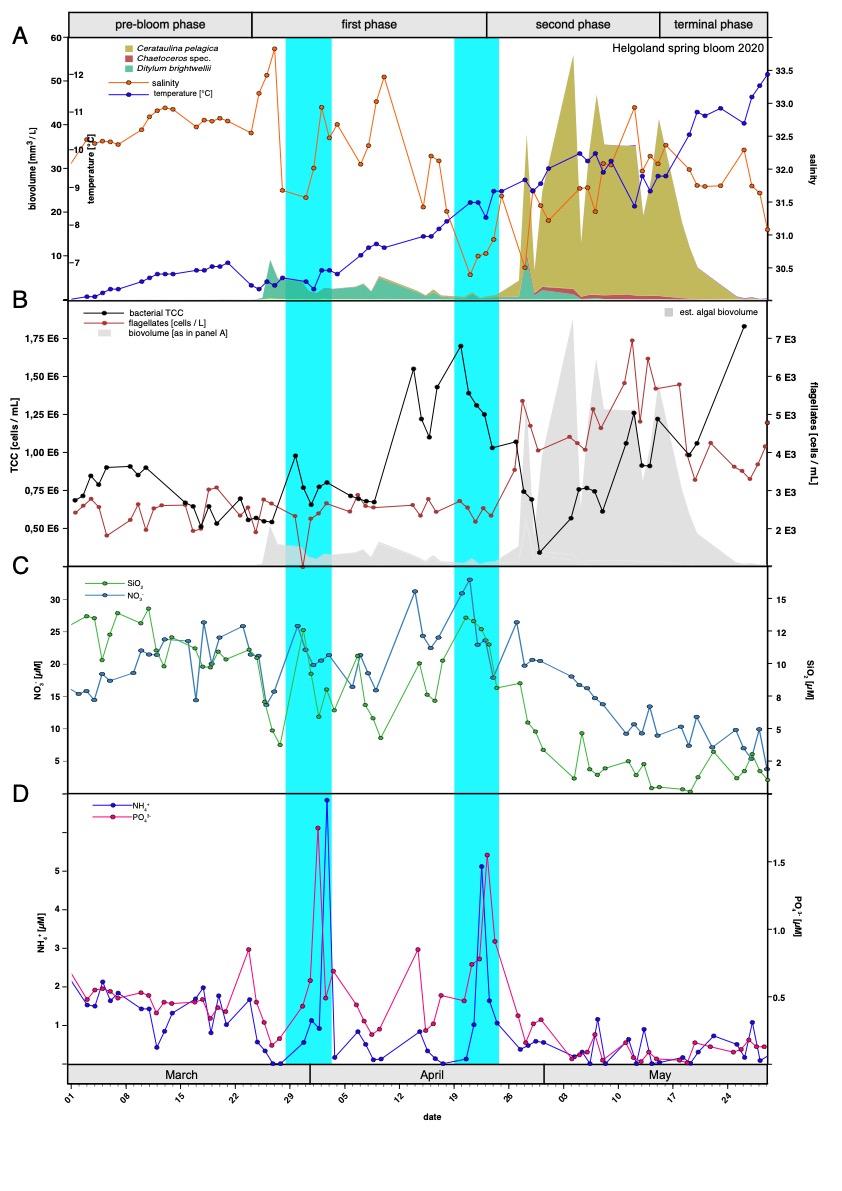
**Fig. S1**. **A.** Levels of salinity and temperature measured. **B.** Total bacterial cell count and flagellates. **C,** **D.** Concentration measured for inorganic nutrients such as silicate, phosphate, nitrate and ammonium over a period of 2020 spring algal bloom.

**Fig. S2.** Wind components at 10 m above the sea surface and obtained from the Climate Data Store of the Copernicus Climate Change Service (ERA 5 product). Corresponding raw data is provided in **Additional file 4**.

**Fig. S3**. Quality of MAGs, **A.** Total number of MAGs obtained at each time-point. MAGs obtained after initial automatic binning are shown in blue color while after manual refinement using anvi-refine are shown in green. Refined MAGs with minimum 70% completeness and maximum 5% contamination are shown in grey bars. **B.** Completeness and contamination score of refined and dereplicated 251 MAGs used in the study. **C.** Assessment of genome size and number of contigs present in final set of dereplicated MAGs.


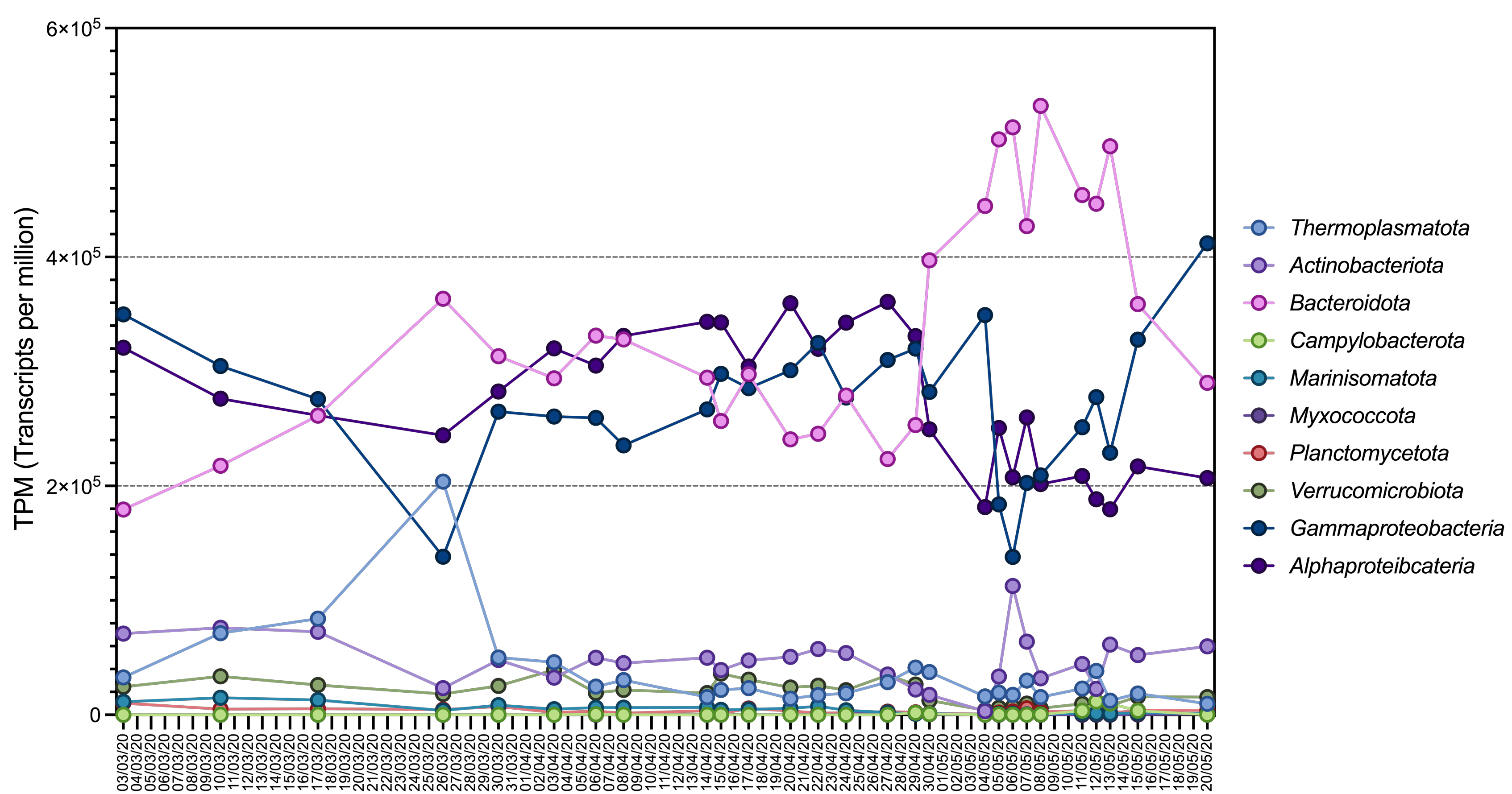


**Fig. S4.** Overall expression of all clades mentioned in terms of transcripts per million during 2020 spring bloom. A clear indication of increase in TPM of *Bacteroidetes* (pink line) during the second bloom phase and decrease in TPM of *Alphaproteobacteria* (dark-blue) was observed.

**Fig. S5.** Transcription pattern of seven archaeal MAGs obtained in this study. All MAGs showed expression during pre-bloom phase. The MGIIa-L1 MAG (W_MB9_1) also showed transcriptional activity throughout the bloom.

**Fig. S6.** Transcriptional profiles of PULs and their predicted polysaccharide substrates in *Aurantivirga* MAG AB_MB169 and C_MB344.

**Fig. S7**. Transcriptional profiles of PULs and their predicted polysaccharide substrates in *Cd.* Prosiliicoccus MAG Q_MB57 and *Polaribacter* MAG X_MB288.

**Fig. S8**. Transcriptional profiles of PULs and their predicted polysaccharide substrates in *Abditibacter* MAG D_MB74 and L_MB280.

**Fig. S9**. Transcriptional profiles of PULs and their predicted polysaccharide substrates in NS4 clade MAG W_MB115.

**Fig. S10**. Transcriptional profiles of PULs and their predicted polysaccharide substrates in all SAR92 clade MAGs: AK_MB88_1, B_MB221, X_MB111, V_MB374, AB_MB236 and SAR86 MAG: P_MB137_1.

**Fig. S11**. Transcriptional profiles of PULs and their predicted polysaccharide substrates in *Luminiphilus* OM60 (NOR5) clade MAGs, AA_MB219 and T_MB10.

**Fig. S12**. Transcriptional profiles of PULs and their predicted polysaccharide substrates in highly expressed and only *Glaciecola* MAG Y_MB157.


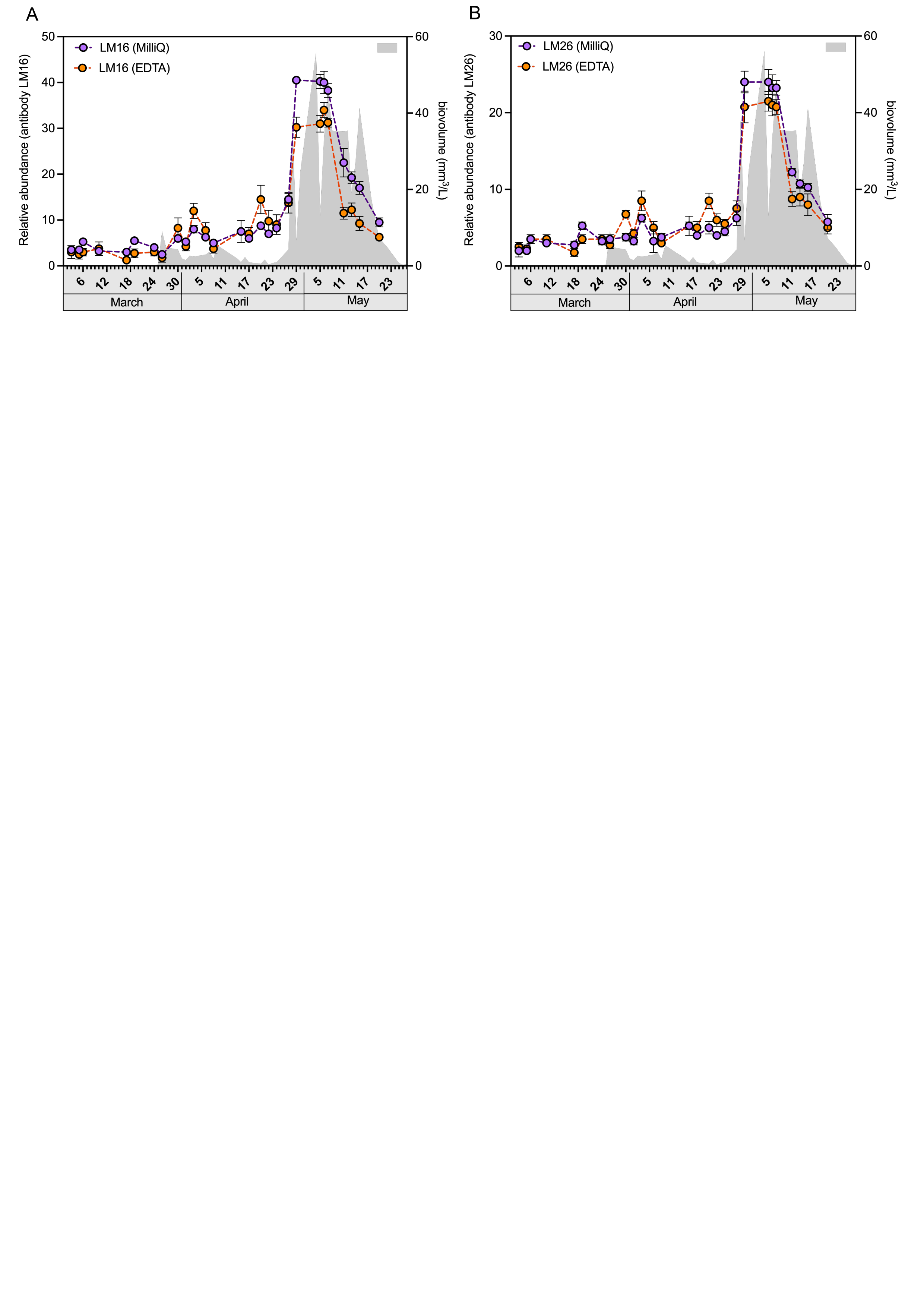


**Fig. S13.** Antibody (LM16, specific for galactosyl residues in rhamnogalacturonan I and LM26, specific for branched (1,6-galactose) (1→4)-β-D-galactan) based measurements of galactose-containing polysaccharides extracted either with MilliQ or EDTA (lines) as compared to the estimated algal biomass data (grey area).


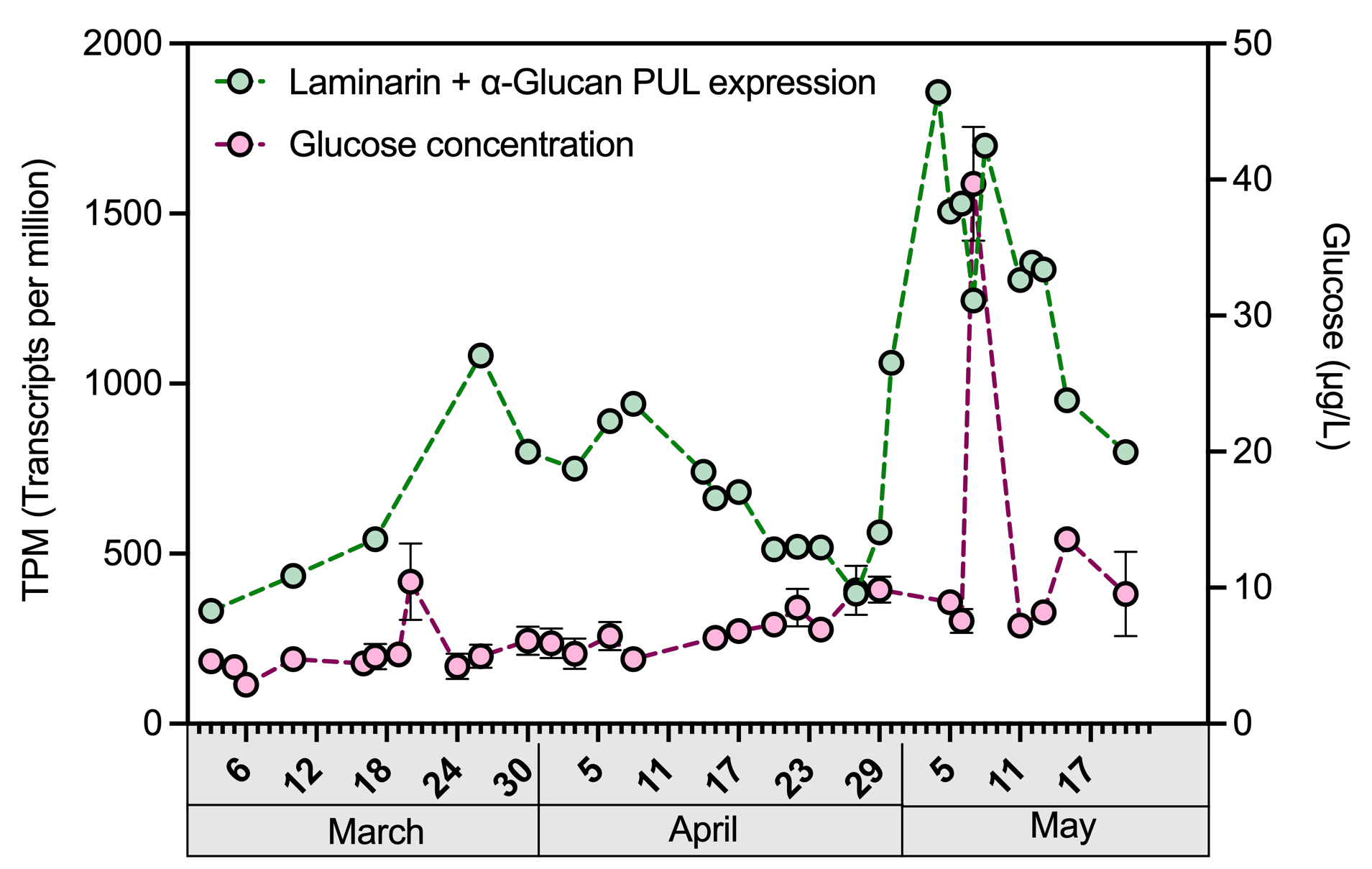


**Fig. S14.** Comparison of the combined expression of PULs targeting laminarin and α-glucan storage polysaccharides that both consist entirely of glucose, and the measured concentrations of glucose in polysaccharides from HMWDOM.


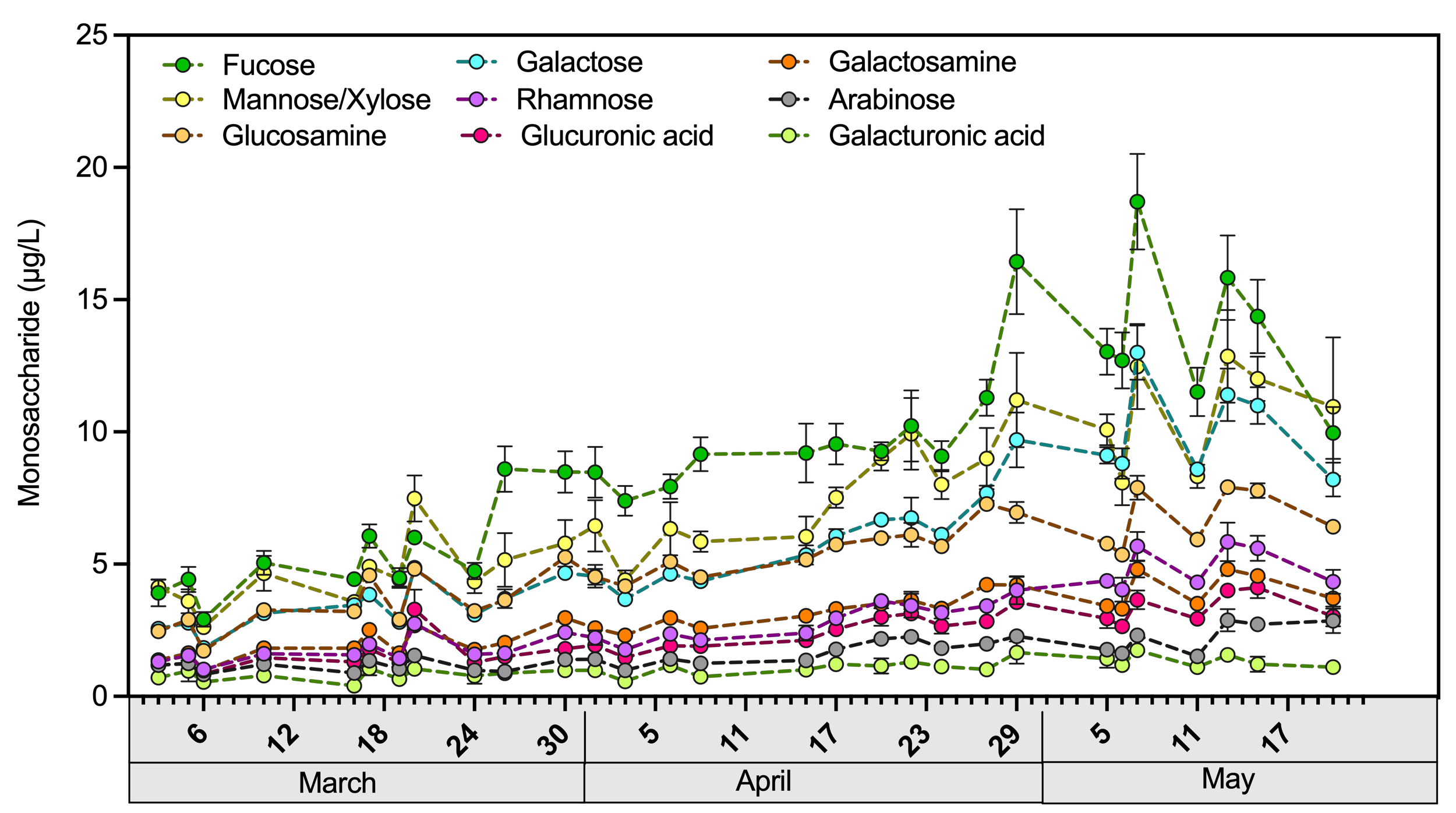


**Fig. S15**. Measured concentrations of monosaccharides other than glucose in HMWDOM across samples. Polysaccharides from HMWDOM were acid hydrolyzed into monosaccharides and quantified by chromatography.

**References**

Avcı, B., Krüger, K., Fuchs, B. M., Teeling, H., & Amann, R. I. (2020). Polysaccharide niche partitioning of distinct *Polaribacter* clades during North Sea spring algal blooms. *ISME J*, *14*(6), 1369-1383.

Engel, A., & Händel, N. (2011). A novel protocol for determining the concentration and composition of sugars in particulate and in high molecular weight dissolved organic matter (HMW-DOM) in seawater. *Mar Chem*, *127*(1-4), 180-191.

Lennon, J. T. (2011). Replication, lies and lesser‐known truths regarding experimental design in environmental microbiology. *Environ Microbiol*, *13*(6), 1383-1386.

Pedersen, H. L., Fangel, J. U., McCleary, B., Ruzanski, C., Rydahl, M. G., Ralet, M. C., Farkas, V., von Schantz, L., Marcus, S. E., Andersen, M. C., Field, R., Ohlin, M., Knox, J. P., Clausen, M. H., & Willats, W. G. (2012). Versatile high resolution oligosaccharide microarrays for plant glycobiology and cell wall research. *J Biol Chem*, *287*(47), 39429–39438.

Vidal-Melgosa, S., Pedersen, H. L., Schückel, J., Arnal, G., Dumon, C., Amby, D. B., Monrad, R. N., Westereng, B., & Willats, W. G. (2015). A new versatile microarray-based method for high throughput screening of carbohydrate-active enzymes. *J Biol Chem*, *290*(14), 9020–9036.

Vidal-Melgosa, S., Sichert, A., Francis, T. B., Bartosik, D., Niggemann, J., Wichels, A. et al. (2021). Diatom fucan polysaccharide precipitates carbon during algal blooms. *Nat Comm*, *12*(1), 1-13.
